# MINDhEARTH: a school-based intervention to improve personal well-being, mindfulness and connectedness to nature in adolescents

**DOI:** 10.3389/fpsyg.2025.1628048

**Published:** 2025-09-08

**Authors:** Francesca Scafuto, Ciro Conversano, Fabio Presaghi, Giulia Santoni, Ilaria Vaglini, Francesco Girardi, Alessio Matiz, Cristiano Crescentini

**Affiliations:** ^1^Department of Languages and Literatures, Communication, Education and Society (DILL), University of Udine, Udine, Italy; ^2^Department of Surgical, Medical, and Molecular Pathology and Critical Care, University of Pisa, Pisa, Italy

**Keywords:** mindfulness, subjective happiness, psychological wellbeing, nature connectedness, acting with awareness, adolescents

## Abstract

The study evaluates the effectiveness of a novel program, called MINDhEARTH, grounded in the ecopsychological approach, and aimed to improve individual wellbeing and ecological self-awareness in adolescents. A pre- and post-intervention design involved 211 students from two Italian high schools, measuring Wellbeing through the Italian version of the Subjective Happiness Scale and Psychological Wellbeing Scale (PWB), Mindfulness through the short-form of Five-Facets Mindfulness Questionnaire and Nature Connectedness through the Italian version of Connectedness to Nature Scale. The questionnaire also included open-ended questions aimed at evaluating qualitative perception of personal benefits from the program. Multilevel regression analysis showed significant effect of intervention by time interaction on Subjective Happiness and on Autonomy, PWB subdimension. Among the facets of Mindfulness, results showed a significant effect for Acting with Awareness and a marginally significant effect for Non-reacting. Contrary to expectations, no significant interaction effect on Nature Connectedness was found. Nevertheless, qualitative reports through qualitative content analysis revealed connection to nature to be the second prevalent theme of perceived benefits, after connection with oneself. The study concludes with analysis of limitations and suggestions for future school mental health programs that consider a combination between mindfulness interventions and nature-based activities to increase wellbeing and antecedents of ecological citizenship.

## Introduction

The current study is aimed at evaluating the effectiveness of a novel intervention in a school setting aimed at increasing self- and ecological awareness. The program combines ecopsychological practices and principles with mindfulness-based activities and was designed to impact psychological and hedonic dimensions of wellbeing, mindfulness, and connectedness to nature in adolescents.

Adolescence is a period of transition with cognitive, affective, and organic transformations that support generativity and the process of individuation, but, at the same time, the risks of psychological problems and improper adaptation increase (Compas et al., 2017). In times of global challenges, such as the climate crisis and rapid social changes, young people seem to absorb the general social distress, highlighting in recent years a reduction in the mental health indexes: globally, one out of seven adolescents from 10 to 19 years suffer from a psychological disorder (Herbst, 2024). In Europe, an increasing number of adolescents report high levels of emotional distress, withdrawal, depression, and anxiety, which lead to a reduction in quality of life (Castelpietra et al., 2022). In Italy, studies revealed this trend has worsened after the COVID-19 pandemic, along with the widespread use of social media and Internet addiction (Ciacchini et al., 2023; Scafuto et al., 2023).

How could we connect the increase of emotional distress and psychological burden with global social and environmental issues? From an ecopsychological perspective, it is particularly compelling to explore this connection since alienation from nature is considered a source of psychological malaise (Chalquist, 2009). According to this view, alienation from nature is so widespread that people are often not aware that their psychological burden may depend on the type of their connection with nature. On the other hand, people who become more aware of this link, may suffer from eco-anxiety since they pay more attention to the environmental signals of the climate crisis and its consequences (Thomson and Roach, 2023). Indeed, almost half of young people aged 18 to 34 (48%) reported stress and eco-anxiety about climate change in their daily lives (American Psychological Association, 2020) while climate change is considered the greatest threat to global health, including mental health, of the 21st century (Costello et al., 2009). The individual struggles that young people face thus seem to reflect the suffering and threats that affect our planet. The hypothesis of a correlation between psychological and planetary wellbeing/distress highlights the need to investigate psychological and socio-psychological variables in order to create psychological interventions, such as those based on mindfulness, that may have a significant impact on young people’s mental health (Cilar et al., 2020) while simultaneously promoting hope and activism (Macy and Brown, 2014).

Mindfulness is defined as ‘the awareness that emerges when we pay attention on purpose, in the present moment, and nonjudgmentally to the unfolding of experience moment by moment’ (Kabat-Zinn, 2003, p. 145). Mindfulness-Based Interventions (MBIs) have been developed, and experimental studies have demonstrated their effectiveness in improving mental health outcomes—such as anxiety, stress, and depression—in both clinical and non-clinical adult populations (Khoury et al., 2015; Haller et al., 2021; Zhang et al., 2021; Kraines et al., 2022).

Beyond the recognized effects of MBIs on mental health in adults, literature insights suggest that MBIs are potentially effective in improving wellbeing as well as reducing psychological distress also among children and adolescents (e.g., Phan et al., 2022). However, the evidence in these populations is mixed, both in Italy (e.g., Crescentini et al., 2016; Feruglio et al., 2022; Scafuto et al., 2024a) and worldwide (Dunning et al., 2022), pointing toward the need for further studies to understand the impact of universal mindfulness programs and the moderator factors to be considered.

### Well-being and mindfulness

Wellbeing is a complex multidimensional construct that Ryan and Deci (2001) have categorized into two general perspectives: hedonic (or Subjective Well-being, SWB) and eudaimonic (or Psychological Well-being, PWB). SWB consists of cognitive (e.g., short-term satisfaction) and affective (e.g., predominance of positive over negative emotions in daily life) components (Diener, 2000), which are more closely associated with immediate pleasure (Bojanowska and Kaczmarek, 2022). In contrast, PWB encompasses various dimensions that reflect a person’s overall mental and emotional health (Ryff and Keyes, 1995). It emphasizes the pursuit of a full and meaningful life rather than merely seeking pleasure or avoiding pain. It also concerns the extent to which an individual is fully functioning (Ryan and Deci, 2001).

The link between mindfulness and wellbeing is highlighted by literature findings. Several meta-analytic studies have provided evidence supporting the beneficial effects of mindfulness practices on various aspects of well-being. MBIs would be effective in both clinical and non-clinical samples, for instance, associated with a defense style as a positive response to psychological distress in professionals (Di Giuseppe et al., 2019) and with the decrease of depression in chronic pain (Veltri et al., 2012). In adolescent populations, McKeering and Hwang (2019) examined the impact of school-based MBIs, finding positive improvements in well-being measures in 11 of 13 studies reviewed. Another meta-analysis revealed that adolescents who practiced mindfulness had lower general distress, depression symptoms, and anxiety (Reangsing et al., 2020). In early adolescents, a mindfulness-based program appeared to be particularly effective in increasing the eudaimonic sense of purpose in life and personal growth with no significant effect on hedonic well-being and psychological distress (Scafuto et al., 2024a).

Through what mechanisms mindfulness would promote well-being has been investigated in several studies that showed MBIs to promote eudaimonic well-being directly (Yeager et al., 2022) and indirectly through, for instance, the increase in emotion regulation strategies (Scafuto et al., 2024b), self-compassion, and cognitive flexibility (Yousefi Afrashteh and Hasani, 2022). According to Hölzel et al. (2011), the mechanisms through which MBIs produce positive effects on well-being can be summarized into four components: attention regulation, body awareness, emotion regulation, and change in the perspective on the self. Attentive awareness is indeed cultivated during MBIs, initially directed toward bodily sensations and subsequently extended to emotions and thoughts. This type of training fosters a perspective of observation regarding the self, enabling individuals to approach emotions with equanimity. Additionally, MBIs promote cognitive defusion by helping individuals detach from static perceptions of the self, thereby supporting greater psychological flexibility and emotional resilience. In general, one of the most relevant theories for the link between MBIs and well-being is the Mindfulness-to-Meaning Theory, which posits that mindfulness fosters positive reappraisal and meaning-making by cultivating a metacognitive state of awareness. Such a state broadens attentional scope, allows individuals to notice new information that can lead to a revised interpretation of everyday life circumstances (Garland et al., 2015).

Alternatively, to above reported findings, two recent large RCT on MBIs at school (Kuyken et al., 2022; Volanen et al., 2020) showed no link between mindfulness and well-being when compared with active controls, raising questions about the role of moderator variables to explain the variability of the programs’ effectiveness. Almost 4 thousand early adolescents (11–16 years) in the UK showed no significant differences between universal mindfulness program and social–emotional learning program on depressive symptoms, socio-emotional-behavioral functioning and well-being at 1-year follow-up (Kuyken et al., 2022). Moreover, in some cases, a worsening of hyperactivity/inattention and emotional problems was revealed (Kuyken et al., 2022). Another large RCT showed a similar result in Finland for early adolescents (Volanen et al., 2020): Mindfulness program compared to Relaxation intervention did not differ for socio-emotional functioning but only for resilience at the completion of the intervention and for depressive symptoms only among girls at 6 months’ follow-up. The authors explained this result considering that programs delivered during school activities may be affected both by the lack of motivation and acceptability of participants, and by the low dose of the program (Kuyken et al., 2022). Indeed, adolescents who carried out their daily independent mindfulness practice demonstrated better resilience at follow-up and improvement in socio-emotional functioning at the completion of the intervention, compared to those who did not attend daily practice (Volanen et al., 2020).

Demographic factors, such as age, were also shown to moderate the link between mindfulness and well-being: MBIs seem to be more effective in late adolescence. In line with this, a study conducted by Gómez-Odriozola and Calvete (2021) has shown that MBI prevented the increase in depression and somatic symptoms, reduced interpersonal difficulties and increased self-esteem in older adolescents; while in younger adolescents there was even an increase in depression and somatic symptoms, with no observable effect on interpersonal difficulties. These age-related differences may be explained by variations in neurocognitive maturity (Johnson et al., 2017), plasticity to change (Roeser and Pinela, 2014) and by the lack of metacognitive skills (Kuyken et al., 2022). Supporting this view, the meta-analysis by Carsley et al. (2018) found that MBIs implemented during late adolescence (ages 15–18) tend to yield greater improvements in mental health and well-being compared to those delivered to younger age groups. Late adolescence, in particular, appears to be a period in which mindfulness is especially beneficial both in the short and long term, as it is considered a ‘window of opportunity’ for cognitive and social development (Roeser and Pinela, 2014).

Although studies exploring gender as a moderating factor are still limited, some studies found that female meditators experienced greater increases in positive affect than male meditators (Kang et al., 2018) and reported higher levels of quality of life (Lassander et al., 2021). A recent RCT suggests that MBI improved depressive symptoms, or low-grade depression, but only among girls and appeared to benefit boys in resilience only after a more regular practice, compared to girls (Volanen et al., 2020).

Could mindfulness skills together with personal well-being increase planetary well-being? Some evidence reveals that eudaimonic well-being is a key factor in understanding the connection between mindfulness and socio-ecological variables. Psychological wellbeing has been investigated as a central factor mediating the relationship between mindfulness and ecological behavior (e.g., Ericson et al., 2014) and influencing changes in value systems (Brown and Kasser, 2005). Indeed, a mindfulness program for adolescents was shown to enhance self-transcendence values—such as universalism and benevolence—through the personal growth dimension of psychological well-being (Scafuto et al., 2025). This finding supports the idea that personal and planetary well-being may be interconnected rather than conflicting (Jacob et al., 2009).

### Connectedness to nature and mindfulness

Connectedness to Nature (CN) is a relatively recent psychological construct that can be considered foundational to the discipline of ecopsychology. Initially, it was defined as the extent to which an individual incorporates nature into their cognitive representation of self (Schultz, 2002), emphasizing the cognitive aspect of identification. Later, it was redefined as an individual’s subjective relationship with the natural world (Mayer and Frantz, 2004), highlighting the importance of emotional and experiential dimensions. Ecopsychologists strongly emphasize the role of CN in fostering eudaimonic well-being, enhancing quality of life (Baceviciene & Jankauskiene, 2022), and promoting pro-environmental behaviors (Martin et al., 2020).

CN seems to be a necessary mediator in the relationship between nature exposition (e.g., experiences in wild and in unpleasant nature and availability of greenspaces in urban contexts) and outcome variables, such as well-being and ecological behavior (Capaldi et al., 2014). Mayer et al. (2009) have shown that nature connectedness mediates the relationship between nature exposure and hedonic well-being. Richardson et al. (2021) showed that engaging with nature through simple activities (e.g., smelling flowers) that enhance the relationship with nature is a stronger predictor of well-being and mental health than merely the amount of time spent in nature.

Regarding the relationship between mindfulness and CN, studies showed a reciprocal and bi-directional influence (Schutte & Malouff, 2018). Positive correlations between nature connectedness, well-being, and mindfulness were found (Howell et al., 2011) as well as significant link between mindfulness and ecology-related variables. For instance, CN has been identified as a mediator in the relationship between mindfulness and sustainable behavior (Barbaro and Pickett, 2016), mindfulness and climate change belief (Wang et al., 2019), as well as between mindfulness and climate change risk perception (in both cognitive and emotional components; Scafuto, 2019).

Not all the facets of mindfulness were shown to predict in the same way the ecology-related variables. The mindfulness facet of *observing* (in the conceptualization of the Five Facet Mindfulness Questionnaire; Baer et al., 2006) has been shown to enhance the perception of climate risk for oneself and the local community, unlike other facets that appear negatively associated with the climate risk perception (Scafuto, 2019, 2021). Observing has also been identified as a strong predictor of ecological behavior (Barbaro and Pickett, 2016) and the only facet correlated with a sense of global identity (Loy and Reese, 2019). Additionally, in fostering ecological behavior other research highlights the relevant role of another key mindfulness facet, i.e., acting with awareness, that is dismissing the “automatic pilot mode” in order to choose what actions to undertake (Amel et al., 2009). In order to explain how mindfulness may promote ecology-related outcomes, the Self-Determination Theory (Brown and Ryan, 2003) could be referred to. Indeed, mindfulness appears to foster autonomy, that is a form of motivation drawing from conscious personal choice and leading to behaviors perceived as self-congruent, enduring overtime, and aligned with one’s core values. This effect of mindfulness is particularly relevant in fostering pro-environmental choices, that usually require high level of autonomy against dominant contextual norms of consumerism and environmental disregard. For example, it was shown that more mindful individuals would eat less meat and have a lower environmental impact associated with their diet (Thiermann and Sheate, 2020), participate more frequently in environmental activism (Wamsler and Brink, 2018), and engage in more pro-environmental behaviors (Jacob et al., 2009; Loy and Reese, 2019). In this way, intrinsic vs. materialistic values, promoted by mindfulness, would affect indicators of pro-environmental behaviors, such as socially conscious shopping, green purchase, and satisfaction with life as well (Ericson et al., 2014). Considering the link between mindfulness and sustainability-related variables, Sheffield et al. (2022), in their review and meta-analysis, underlined how nature-based interventions may increase connectedness to nature over time, maintaining the effect in the follow-up measures.

An example of nature-based intervention that combined mindfulness with sustainability is the so-called Mindful Climate Action (MCA), carried out by Grabow et al. (2018) and inspired by Barrett et al. (2016). The eight-week education program merges contents on energy use, climate change, and sustainability with training in mindfulness meditation. The program is based on the structure of a standard Mindfulness-Based Stress Reduction program (MBSR) together with a group discussion about climate issues. In their study, the authors successfully demonstrated the feasibility of the program and estimated a reduction in participants’ individual carbon footprints in areas such as food, transportation, and household energy (Grabow et al., 2018). However, the program was limited to adults and included only a small number of participants.

Another well-known example of an intervention to increase ecological self-awareness is “The Work That Reconnects,” developed by Macy and Brown (2014), drawing from Systems Thinking, contemplative practices and Deep Ecology. This approach has especially inspired the MINDhEARTH program that is presented here. The work includes a choice among over 60 activities designed to foster nature connectedness through a spiraling process. The process encompasses four phases: 1. (Gratitude); 2. (Honoring the pain); 3. (Seeing with new eyes); 4. (Going forth). In the first phase, experiential activities are aimed at increasing grounding through a feeling of gratitude for present and past places, people, and living beings one is related to. In Honoring the Pain for the World, activities are thought to increase emotional sensitivity, expression and sharing of pain, sadness or anger for global suffering. What had isolated individuals in private anguish at this stage opens outward increasing a sense of interdependence (Macy and Brown, 2014). This allows Seeing with New Eyes that are cognitive frames of no separation between humans and environment, between social and environmental justice; past, present, and future generations. In Going Forth, individuals move into actions according to their skills and limitations, designing projects with others whenever possible.

In addition, the so-called Gaia program was also aimed to increase both individual and ecological self-awareness through mindfulness, devoting its last modules for knowing the charter of earth and guided meditations, such as *being a tree* or *being a planet* (Ghiroldi et al., 2020). It was applied in the school context with children and adolescents, showing effects on wellbeing and self-transcendence values through the dimension of psychological wellbeing, that is personal growth (Scafuto et al., 2025).

The combination between mindfulness and nature-based exercises is not just aimed at increasing ecological self-awareness but it also affects personal well-being. Experimental studies showed that nature exposure together with mindfulness was more effective in increasing well-being than stand-alone practices such as nature exposure and experience in nature (Unsworth et al., 2016), as well as mindfulness-based intervention alone (Nisbet et al., 2019). Similarly, according to Choe et al. (2020) the widely employed MBSR program has greater and more lasting effects on subjective well-being and mental health, when experienced in a natural outdoor environment, compared to an indoor or built environment.

The meeting point between mindfulness and nature can be referred to by the term “green mindfulness” that identifies an approach aimed at developing the awareness necessary “to expand the boundaries of one’s individual identity toward a sense of co-participation in the natural world” (Danon, 2020; p. 64) and to develop ecological citizenship (Widening), personal leadership (Centering), and ecological relations (Tuning; Danon, 2020).

In comparison to mindfulness meditation, which primarily focuses on internal stimuli such as on bodily sensations and/or thoughts and feelings, exposure to nature within the framework of green mindfulness may imply, instead, that the focus of attention is more outwards than inwards. Moreover, while traditional mindfulness requires active practice and training to cultivate open attention, mindfulness practiced in nature may require a reduced attentional effort (Lymeus et al., 2018). This effect can be explained by Kaplan’s Attention Restoration Theory (1995), which posits that the regeneration of directed attention occurs through involuntary attention or fascination (a “passive” approach that does not require inhibition and control mechanisms) and is favored by exposure to natural environments (Hartig et al., 1991). Experiencing nature is often easier because it distances individuals from daily routines, modifies human perception of time and space, aligns individual inclinations and environmental opportunities, and allows for fascination (Barbiero and Berto, 2016). However, the mere experience of nature may not be sufficient to activate open attention if there has not been prior education aimed at developing one’s biophilia, that is, the innate tendency to focus attention on living systems and to form emotional affiliation with them (Wilson, 2002).

Given the severity of the climate crisis and its wide-ranging socio-psychological implications, it is surprising that there remains a lack of interventions designed to enhance both individual and planetary well-being, beyond purely environmental educational program based on increasing knowledge on the implications of human lifestyle on the climate or beyond the abovementioned programs. Findings support the idea that even when people are informed and concerned about climate change, they may feel paralyzed or useless when it comes to acting (Landry et al., 2018). The underpinned idea of the current study is that social and environmental change can be, instead, driven by self-awareness and mindfulness (O’Brien, 2022). Considering the promising results of mindfulness and ecopsychological practices rooted in nature connectedness, both approaches could be integrated to achieve both individual and socio-psychological benefits.

In sum, nature-based mindfulness intervention has received a recent growing attention in research and recent studies showed positive impacts to well-being (e.g., Choe et al., 2020; Lymeus et al., 2018) in adults or emerging adults. Nevertheless, there is still a lack of research and interventions, above all, on youth, therefore this study would play a key role in addressing the research gap in applying nature-based mindfulness programs in adolescents.

### The current study

The present study aimed to evaluate the effects of a novel mindfulness-based program, MINDhEARTH (see Table 1), which incorporates activities designed to foster ecological self-awareness and builds upon previous similar programs (Scafuto et al., 2022, 2024a, 2024b). Specifically, the study examined its impact on both psychological and hedonic well-being, as well as connectedness to nature in adolescents.

**Table 1:** MINDhEARTH program.

Modules	Specific objectives	Themes	Activities	Duration in sessions
Motivation	Introduction: Purpose and Practices of the Program. Eliciting Individual Needs, Curiosities, and Expectations for the Program.	Well-being: What promotes it and what not; Connection between individual well-being/ill-being and that of the planet. Climate crisis: As an awareness crisis; As an expression of powerlessness.	Sharing the common pact as a guide for the meetings. Pre-test administration	1
MindBody	Eliciting Attention and Awareness: Focus on the breath and bodily activation levels through the engagement of the sympathetic and parasympathetic nervous systems.	Stress: implication mind–body connection Reactions to it (managing the transition from hyperarousal to hypo-arousal) Inhibition of action	Gentle and intense energy exercises (Practices for activating the sympathetic and parasympathetic nervous system), Breath-focused meditation, Body scan: head, chest, abdomen.	1
MindEmotion	Promoting Psychological Awareness: Identifying Tensions and Action Blockages, and Expressing Connections between Sensations, Emotions, and Thoughts.	Psychological Awareness: Living in the present moment Exploring attachment/fusion with thoughts and emotions and letting them go (non-judgmental observation of internal states)	Body-scan exercises, Psychosomatic drawing.	2
MindCommunity	Cultivating Gratitude and Attention toward the positive aspects of relationships with others and emotional resonance and syntonisation.	Relationships: How relational blocks are generated (conflicts and rejections) How to reconnect with others	Practices of gratitude and compassion toward oneself and others, Paired movement and breathing exercises, Deep listening without interruptions.	2
MindNature	Developing a sense of belonging to nature, as ‘I am nature’. Engaging with the unpleasant internal experiences arising from disconnection with nature. Developing a sense of agency that can promote personal empowerment and commitment to small meaningful ecological actions.	Sense of belonging to nature: human-nature analogy, emotions related to the climate crisis. Engaged ecological action: individual or cooperative	Activities in direct contact with nature (green mindfulness, e.g., guided mindfulness walks in nature); Activities in indirect contact with nature (nature sounds; image cards depicting the impacts of the climate crisis and testimonies of activism; videos on Earth conservation).	2
MindAction	Activating visionary and creative problem-solving to address issues related to the climate crisis. Increasing awareness of daily consumption choices and ecological impact. Enhancing sense of efficacy	Ecological awareness Self-efficacy Empowerment	Future visioning exercises (e.g., imagining an accomplished ecological transition, a society that has resolved the climate crisis); Designing micro ecological actions.	1

The first hypothesis is consistent with previous findings demonstrating the positive effects of mindfulness-based interventions on adolescents’ well-being. We hypothesized that the program would primarily enhance the eudaimonic dimensions of well-being, specifically purpose in life and personal growth, in line with a previous study (Scafuto et al., 2023). However, no specific hypothesis is formulated regarding hedonic well-being, given that in previous literature MBIs were shown to have more controversial effects on hedonic dimensions of wellbeing (Reangsing et al., 2020; Scafuto et al., 2023). The second hypothesis concerns the effectiveness of the program in improving mindfulness skills. Since the intervention integrates both mindfulness and ecopsychological practices, it is expected to enhance the mindfulness facets particularly relevant to ecological-related outcomes, such as observing (Barbaro and Pickett, 2016) and acting with awareness (Amel et al., 2009).

Finally, given the program’s emphasis on relational experiences with nature (see Table 1, last two modules), which encourage sensory openness, open attention to the natural environment, and empathy for the consequences of climate change, our third hypothesis predicts that the program will also improve connectedness to nature. This hypothesis is along with other literature that identifies mindfulness as fostering a better relatedness to nature (Scafuto, 2019), that in turn would increase the benefits from nature exposure (Chang et al., 2024).

Regarding socio-demographic variables, we hypothesized that age may have a significant co-variate role related to the outcomes. We hypothesized that older adolescents may benefit more from the program, thus increasing more eudaimonic wellbeing, mindfulness, and connectedness to nature, than younger teenagers. This hypothesis is in line with previous results (Carsley et al., 2018; Gómez-Odriozola and Calvete, 2021) that identify how mindfulness programs seem to increase more mental health and well-being in older adolescents and adults, than in early adolescents. Regarding gender, along with uncertain previous findings (Kang et al., 2018; Lassander et al., 2021; Volanen et al., 2020), we did not formulate specific hypotheses, but we also controlled this variable in the multilevel model. Another purpose of the current study was to explore qualitatively the type of perceived benefits of the program. The idea was to investigate what specific benefits were felt by participants and if they encompassed the three main themes of the program: self-, social, and ecological awareness.

## Methods

### Participants

*A priori* power analysis was used to determine optimal sample size. By planning to recruit at least 80 participants per condition grouped in at least 5 different school classes and setting the expected effect size to 0.10 for each outcome (i.e., the expected change in the Intervention group at T1 in comparison with the Control group), the estimated power for this sample size was about 1-*β* = 0.80 (95% C. I.; 0.79; 0.82), assuming a probability of type I error of *α* = 0.05, a within residual variance of 0.3, and a between classes random variance of 0.3. Statistical power and the optimal sample size were estimated using a Monte Carlo approach with 5,000 sample draws (Kleinman et al., 2021).

A total of 211 participants completed the study, with an average age of 15.29 years (SD = 1.11; Min-Max = 13–18). The sample included 78 male participants (M age = 15.05, SD = 0.99) and the remaining were female (M age = 15.46, SD = 1.15). Participants were recruited from two high schools in the Tuscany region of Italy: one specializing in human sciences and the other in computer science. Table 2 presents the distribution of participants assigned to the Control and Intervention conditions based on their school classes. Regarding the number of students for class, descriptive statistics showed a general average of M = 17.6 (DS = 3.5), with Control showing M = 18.17 (DS = 2.6) while Intervention group showing M = 17.0 (DS = 4.3). Classes included students with Specific Learning Disorders (who have the same learning objectives of the class but at a lower level, e.g., Dyslexia), some with certified disabilities (who have different learning objectives, e.g., ASD, ADHD or cognitive delay) and students with other special needs (who do not have any medical certification but are supported by teachers through compensatory and dispensatory instruments, e.g., students with psychological and socio-economic challenges that interfere with their learning). In accordance with support teachers, the students with certified disabilities were present in the class and at their own pace participated in the activities. Among these students, only two were excluded by the research, owing to the seriousness of language and communication impairment due to their disability (late stage of ASD). The students with learning disorders and with not certified special needs also participated in the intervention, but for privacy reasons of the school policies, we did not collect individual data on students’ learning disorders, disabilities or other special needs or any sensitive data that could prevent anonymity. Indeed, all questionnaires were completed anonymously, as specified in the informed consent signed by the parents. Neither students nor their parents refused to participate in the program to attend the regular class lessons with the teachers.

**Table 2:** Frequencies of participants as function of Intervention groups and of school classes.

Groups	School classes				Total
	1st year high school	2nd year high school	3rd year high school	4th year high school	
Control	38 18%	38 18%	20 9.5%	13 6.2%	109 51.7%
Treatment	32 15.2%	37 17.5%	13 6.2%	20 9.5%	102 48.4%
*Total*	70 33.2%	75 35.5%	33 15.6%	33 15.6%	211 100%

χ^2^ = 3.269; df = 3; Cramer’s V = 0.124; *p* = 0.352.

### Procedure and design

**Figure 1:**
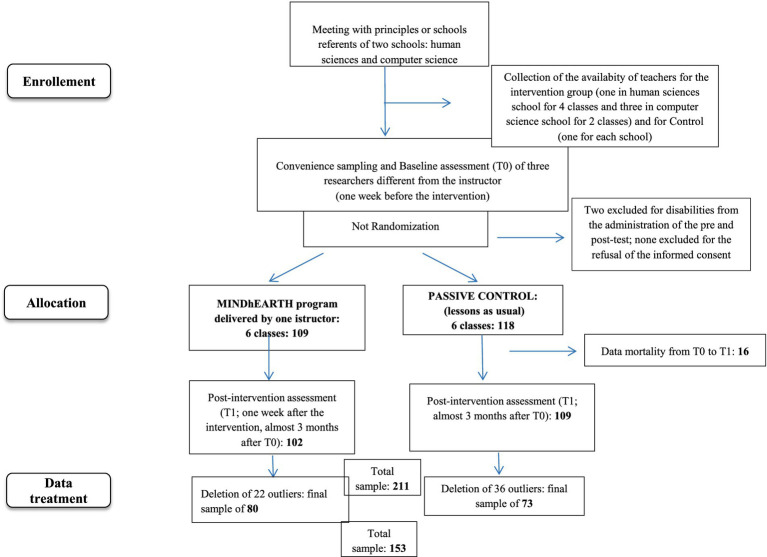
Flowchart of study procedure. The two schools are “G. Carducci” of Pisa (Human Sciences) and “G. Marconi” of Pontedera (Computer Science).

The study followed a no-randomized pre-post-controlled design conducted with embedded mixed methods, that include the prevalence of quantitative measures on the qualitative measures that provide deeper explanation of quantitative data. The intervention was delivered by one instructor, F. S., a researcher and psychotherapist, instructor of mindfulness, who was independent from the researchers who administered pre- and post-test assessments (G. S., F. G.) and from the researcher who analyzed the qualitative reports (F. G.). To evaluate the effects of the MINDhEARTH program, six classes were no-randomly assigned to the Intervention group (*N* = 6 classes; 109 subjects), that received the MINDhEARTH program, and the remaining six to the Control group (*N* = 6 classes; 118 subjects), paired with the former for the type of studied subjects, school, and schedule (Figure 1). Two subjects with serious ASD were excluded from the research and did not fill in pre- and post-questionnaires.

Group assignment was based on teachers’ availability to allocate time for the expert to deliver the program. After the approval of the project by the teachers’ committee and by the principle, some teachers voluntarily offered their lesson time to allow the external expert to deliver the project at school. These classes became experimental ones, and parallel classes for subjects and lessons schedule were then selected as control classes. As a result, randomization was not feasible for either sampling (i.e., a convenience sample) or group assignment. After the signature of parents’ informed consent, measures were taken at two time points: in the Intervention group, data were collected pre-post training (at T0, 1 week before the beginning, and at T1, 1 week after the end of the program); in the Control group, data were collected in two temporally matched sessions. The group assigned to the MINDhEARTH program (i.e., Intervention group) included 102 participants while the control group included 108 students, after considering the removal of 16 students (7 from Intervention Group and 9 from Control Group) for data mortality from pre-test to post-test. Hence, the sample decreased from 227 to 211 adolescents. In the Intervention group, the program was conducted during classroom lessons; in the Control group, the students attended usual classes during the study. All students received information about the general aims of the study. The research was conducted from November 2023 to June 2024. The study was approved by the Ethics Committee for the psychological research of the University of Pisa (No. 0043108 in 13/2023).

### The MINDhEARTH program

The MINDhEARTH program is a mindfulness-based program which focuses on body–mind interaction (the second and third module, after a first module of motivation) and on the improvement of social (the fourth module) and ecological self-awareness (the fifth and six modules; see Table 1). The program was mainly developed by F. S., and delivered by the same, who has been an instructor of psychosomatic mindfulness (Ghiroldi et al., 2020; Quinto et al., 2025) and psychotherapist for adolescents for several years. It was inspired by an already investigated program, the Gaia program, described in previous studies (e.g., Ghiroldi et al., 2020), and by the Work that Reconnects (Macy and Brown, 2014). It included 9 two-hour weekly sessions (18 h), delivered at schools and in outdoor settings (i.e., beaches, public gardens, school gardens). Unlike previous interventions of the authors (Scafuto, 2021; Scafuto et al., 2023; Scafuto et al., 2022), MINDhEARTH explicitly incorporates ecopsychological practices aimed at enhancing ecological self-awareness.

The structure of each session included an introduction to the themes using facilitation tools for dialog, interactive games, guided practices (e.g., mindfulness body scans, bodily exercises to reduce allostatic load, and ecopsychological practices), and a final sharing session in pairs and in the group circle. The program consisted of six modules focusing on various aspects of mindfulness and personal and planetary well-being (motivation, bodily self-awareness, emotions, community, nature, action). In the first module, participants were introduced to the program, their needs and questions were collected and reflections and personal memories of experiences about well-being were shared. The second module was focused on perception of stress in the body arousal, involved practices to deal with stress through the balance of sympathetic and parasympathetic system, mindfulness practices focused on breath, sensations toward external stimuli (such as flowers, herbs), and deep relaxation. The third module focused on the connection between emotions and interoceptive feelings of body tensions through techniques such as body scan of tensions/sensations of constraints. The fourth module regarded the way to engage in stressful or healthy social relationships. Participants were helped to enter a mindful space through listening to the needs of others and finding a common rhythm, starting from body connection (e.g., eye contact, hands’ dance movement) and practices of gratitude. The fifth module introduced the experience of sensory opening in nature, guided meditation on the analogies between humans and nature, and sharing of information on the issue of climate crisis with personal practical implications and expression of emotions linked to this knowledge, whereas, the sixth module fostered engagement in action through practices to reinforce a sense of self efficacy for planetary and personal well-being, identifying what individual and collective subsequent actions to undertake, through creative and visioning activities about future. All the six modules with specific aims, themes, and activities are fully described in the appendix (Table 1). Every session lasted 2 h with a break of 10 min. The session was not entirely of mindfulness (it usually lasted almost 20 min) as it included an initial dialog to prepare the theme, ice-breaking games to improve social connection, and increase motivation, then the main practices of mindfulness and nature-based practices. Finally, the participants shared in pairs and in a circle through deep listening modalities. The program was delivered mostly in the school classes, with just a few sessions of the program outdoors depending on what was permitted.

### Measures

To verify our hypotheses about the effectiveness of the program, we focused on measures of hedonic and eudaimonic psychological wellbeing, mindfulness abilities, and connectedness to nature. To assess the reliability, we analyzed McDonald’s *ω* from the current study dataset which are reported as follows.

- To measure eudaimonic wellbeing, the 18-item validated Italian version of the Psychological Wellbeing Scales (PWB, Sirigatti et al., 2013) was used. The scale is composed of six subdimensions, each of which contains three items: Self-Acceptance (a positive attitude toward the self and one’s past life); Autonomy (self-regulation and independence); Environmental Mastery (the competence to manage the environment and external activities); Personal Growth (positive attitude to new experiences); Positive Relations with others (the ability to have an open and satisfying relationship with others); Purpose in Life (believing that there is a meaning to one’s life). The reliability for PWB subdimensions was adequate: Personal Growth (McDonald’s ω, at T0 = 0.68; at T1 = 0.84), Self-Acceptance (McDonald’s ω, at T0 = 0.83; at T1 = 0.85), Positive Relationship with Others (McDonald’s ω, at T0 = 0.69; at T1 = 0.66), Environmental Mastery (McDonald’s ω, at T0 = 0.66; at T1 = 0.70), and Purpose in Life (McDonald’s ω, at T0 = 0.67; at T1 = 0.66). The only subdimension that showed lower reliability was Autonomy (McDonald’s ω, at T0 = 0.48; at T1 = 0.47). In the PWB scale, items are rated on a six-point Likert scale (1 = *strongly disagree*, 6 = *strongly agree*), with higher scores indicating higher eudaimonic wellbeing. To evaluate hedonic well-being, Subjective Happiness Scale (SHS; Lyubomirsky and Lepper, 1999) assessed the individual evaluation about one’s own happiness also in comparison to others and was used in its Italian version including four items (Iani et al., 2013; McDonald’s ω, at T0 = 0.78; at T1 = 0.82), rated on a six-point Likert scale (from 1 = *a not very happy person/ not at all* to 6 = *a very happy person/very much*) with higher scores indicating higher hedonic wellbeing. To evaluate Connectedness to Nature, that reflects an individual’s subjective relationship with the natural world (Mayer and Frantz, 2004), the Connectedness to Nature Scale (CNS) in its Italian version (Di Fabio and Bucci, 2016) was used (McDonald’s ω, at T0 = 0.83; at T1 = 0.87). The scale is composed of 14 items with response options on a 5-point Likert scale ranging from 1 = *very false* to 5 = *very true*.
- Mindfulness skills were assessed with the 15-item Short-form Five-Facet Mindfulness Questionnaire (FFMQ; Baer et al., 2012) in its Italian version (Giovannini et al., 2014). The five mindfulness facets were: Observing, that is the experience of inner and outer stimuli such as sensations, cognitions, emotions (McDonald’s ω, at T0 = 0.58; at T1 = 0.62); Non-reacting, that is the ability to allow thoughts and feelings to come and go (McDonald’s ω, at T0 = 0.62; at T1 = 0.64); Acting with awareness, that is the skill to leave the automatic pilot mode of life (McDonald’s ω, at T0 = 0.72; at T1 = 0.74); Non-judging, that is the ability to take a nonjudgmental stance toward one’s experience (McDonald’s ω, at T0 = 0.76; at T1 = 0.78); Describing, that is the ability to express internal experiences through words (McDonald’s ω, at T0 = 0.60; at T1 = 0.62). Participants were questioned on what extent each item was true for them on a 5-point Likert scale ranging from 1 (*Never or Very Rarely True*) to 5 (*Very Often or Always True*). Higher values of the scales indicated higher mindfulness.
- The agreeability of the program was evaluated with two questions: 1. “How pleasant was your involvement in the program?” 2. “How much were you able to overcome the difficulties encountered during the program?” The answers were given on a 5-point Likert scale ranging from 1 (*Not at all*) to 5 (*Very much*).
- The perceived benefits of the program were evaluated with an open-ended question at the end of the questionnaire: “If and in what do you already feel the benefits of the program?,” to which the same sample of the students had to briefly answer. Hence, 102 people of the experimental group also responded to this question, six among these answers were excluded because they were either missing or blank spaces, thus 96 answers were processed using a qualitative content analysis (QCA; Schreier, 2012).

### Analysis strategy

Preliminary analyses were conducted to verify the normality of the variables: none of the outcome variables showed skewness and kurtosis above 4. By checking multivariate normality (Henze-Zirkler HZ = 1.00, *p* < 0.01), 58 participants were identified as multivariate outliers and were excluded from the analysis. After outlier deletion, participants in the non-Intervention group were *N* = 73 while participants in the Intervention condition were *N* = 80. Regarding the missing values, we replaced the missing ones with the mean value for those outcome variables, making 20 replacements for the Intervention Group and 12 for the Control Group.

To test our research hypotheses, we run a multilevel regression model for each relevant outcome (Subjective Happiness, Nature Connectedness, the six subdimensions of PWB, the five facets of the FFMQ), with the following set of predictors: a factor coding the time conditions (Pre- vs. Post-test), a factor coding the Intervention conditions (Control vs. Intervention) and an interaction factor between time and intervention. We also covaried the effects of gender and age. The hierarchical structure of data accounted for the clustering of time (pre-test and post-test measures), within participants, and within school classes. Finally, to test the cross-level interaction between time and intervention (to test the efficacy of the MINDhEARTH intervention), we also added the random effects for intercept and for the time variable. We used Markov Chain Monte Carlo (MCMC) estimation method for this analysis (with “burn-in” length ranging from 1,000 to 3,000 followed by 50,000 iterations and initial values provided using RIGLS) to account for dependency between repeated observations. All multilevel analyses were run with the MlWin software (Rasbash et al., 2009) in R software (R Core Team, 2022) by using the library R2MLwiN (Zhang et al., 2016).

To analyze the qualitative data, that is the answer to the open-ended question about perceived benefits, we performed a qualitative content analysis (QCA). QCA is a method for generating a systematic description of qualitative data by classifying the meaning of the material in a coding frame. Just one coder was involved, with the supervision on the process of analysis of the principal investigator (F. S.), nevertheless no analysis was carried out to evaluate the inter-coder reliability and cross-checking. We followed the eight steps for QCA indicated by Schreier (2012). Three steps are in common with all the methods: deciding the research question, selecting the material and the final step of interpretation and presentation of results. We briefly describe the specific steps of the analysis. The third step was: building a coding frame that comprised main categories (here called “themes”) and subcategories (here called “categories”). For the fourth step, the material was divided into units of coding that in the fifth step were tried out through double-coding. The sixth step was the discussion of units that were ambiguous, thanks to the confrontation with a second researcher and the revision of the coding frame. The seventh step was the main analysis, hence the application of the revised frame to the entire material. In our case, every answer was considered and codified, regardless of frequency of the identified new concepts. The process did not need a complex frame since the answers analyzed were already very compact and synthetic. Finally, we interpreted and presented the findings through public meetings to the students and teachers of the schools and through this paper (eight step).

## Results

### Descriptive statistics and baseline equivalence

Table 3 shows descriptive statistics (M and SD) at pre- and post-test as function of the intervention condition (Intervention *n* = 80; Control *n* = 73) for the outcome variables. Descriptive data organized by time, intervention, and class are shown in Supplementary Table S1.

**Table 3:** Descriptive statistics (M and SD) at pre- and post-test as function of the intervention condition for the outcome variables.

Variables	Pre-test				Post-test			
	Control		Intervention		Control		Intervention	
	M	SD	M	SD	M	SD	M	SD
Subjective Happiness (SH)	3.83	1.00	3.85	0.98	3.76	0.94	4.09	0.99
Nature Connectedness (CNS)	3.10	0.53	2.88	0.60	3.14	0.53	3.05	0.66
FFMQ Observing	2.60	0.90	2.42	0.92	2.75	0.89	2.46	0.97
FFMQ Describing	2.67	0.72	2.77	0.82	2.70	0.76	2.93	0.74
FFMQ Acting	3.59	0.88	3.72	0.88	3.35	0.81	3.87	0.78
FFMQ Non-judging	3.28	1.00	3.40	0.99	3.37	0.81	3.75	0.97
FFMQ Non-reacting	2.53	0.82	2.53	0.82	2.56	0.74	2.29	0.81
PWB Self-Acceptance	3.71	0.99	3.52	1.06	3.68	0.95	3.62	1.15
PWB Autonomy	4.09	0.81	3.93	0.96	3.98	0.82	4.11	0.78
PWB Environmental Mastery	3.63	0.95	3.76	0.91	3.78	0.92	3.84	0.82
PWB Personal Growth	4.38	0.80	4.15	1.04	4.40	0.92	4.00	1.17
PWB Positive Relations	4.20	1.06	4.03	1.08	4.09	0.95	4.09	0.97
PWB Purpose in Life	3.57	0.92	3.58	0.93	3.39	1.01	3.66	0.89

By checking for significant differences between the two comparison groups at pre-test, we run a series of ANOVA with fixed (Intervention) and random effects (random intercept) on the outcomes considered in the present study. We did not find significant differences but for Nature Connectedness [*F* (1, 150.96) = 6.00, *p* = 0.02; see Table 4]. In particular, comparing the means of the two groups at the pre-test, the control group scored higher on CNS than the intervention group.

**Table 4:** Baseline equivalence.

Variables	*DF*	*F*	*p*
Subjective Happiness (SH)	1, 151.00	0.02	0.88
Nature Connectedness (NC)	1, 150.96	6.00	0.02
FFMQ Observing	1, 150.44	1.60	0.21
FFMQ Describing	1, 150.41	0.55	0.46
FFMQ Acting	1, 150.55	0.88	0.35
FFMQ Non-judging	1, 150.96	0.37	0.54
FFMQ Non-reacting	1, 150.48	0.00	1.00
PWB Self-Acceptance	1, 151.00	1.34	0.25
PWB Autonomy	1, 151.00	1.32	0.25
PWB Environmental Mastery	1, 151.00	0.73	0.39
PWB Personal Growth	1, 150.51	2.27	0.13
PWB Positive Relations	1, 149.35	0.97	0.33
PWB Purpose in Life	1, 150.99	0.10	0.76

### Intervention effects on wellbeing, mindfulness, and nature connectedness

Concerning the effects of the MINDhEARTH program on hedonic well-being (SHS, Table 5), we found no direct significant effects for time (b = −0.032, s.e. = 0.048, *p* = 0.496, Bayesian 95% Cred. Interv. = − 0.125; 0.061) and for intervention (b = 0.134, s.e. = 0.159, *p* = 0.398, Bayesian 95% Cred. Interv. = − 0.18; 0.444), while we found a significant effect for intervention by time interaction (b = 0.152, s.e. = 0.069, *p* = 0.027, Bayesian 95% Cred. Interv. = 0.015; 0.287). Simple slopes analysis showed that for the control group, the effect was negative and non-significant (b = −0.03, s.e. = 0.05, *p* = 0.48) while for the intervention group, the effect was positive and significant (b = 0.12, s.e. = 0.05, *p* < 0.01). Considering random effects, we found significant differences among school classes (L3 σ^2^ = 0.018, SE = 0.046, Bayesian 95% Cred. Interv.: 0.001; 0.106) and among students (L2 σ^2^ = 0.625, SE = 0.098, Bayesian 95% Cred. Interv.: 0.452; 0.836), but not among time observations.

**Table 5:** Intervention efficacy for Subjective Happiness.

		*b*	*s.e.*	*p*	*L. L. 95% Cred. Int.*	*U. L. 95% Cred. Int.*
Fixed effects	Constant	3.978	0.323	<0.001	3.341	4.607
	Intervention	0.134	0.159	0.398	−0.180	0.444
	Time	−0.032	0.048	0.496	−0.125	0.061
	Gender (Female)	−0.102	0.170	0.548	−0.436	0.231
	Age	0.090	0.085	0.287	−0.073	0.261
	Intervention*Time	0.152	0.069	0.027	0.015	0.287
Random effects	L3-Classes: Constant	0.018	0.046		0.001	0.106
	L2-Students: Constant	0.625	0.098		0.452	0.836
	L1-Time: Constant	1.195	1.394		−2.102	3.517
	L1-Time: Constant*Time	−0.012	0.033		−0.076	0.053
	L1-Time: Time	−0.834	1.398		−3.163	2.469

Model Fit D-bar = 546.77; L. L. 95% Cred. Int., Lower Level Bayesian 95% Credible Interval; U. L. 95% Cred. Int., Upper Level Bayesian 95% Credible Interval.

Concerning the effects of the program on the sub-dimensions of psychological well-being (supplementary Tables S2–S6), we found a significant effect of the intervention by time interaction on Autonomy (b = 0.151, SE = 0.077, *p* = 0.049, Bayesian 95% Cred. Interv.: 0.003; 0.310, Table 6). Simple slope analysis showed a negative and non-significant linear trend for the Control group (b = −0.06, s.e. = 0.05, *p* = 0.26), and a positive and non-significant trend for the Intervention group (b = 0.09, s.e. = 0.05, *p* = 0.1). Among random effects, we found significant variability among school classes (L3 σ^2^ = 0.017, SE = 0.04, Bayesian 95% Cred. Interv.: 0.001; 0.101) and among students (L2 σ^2^ = 0.273, SE = 0.065, Bayesian 95% Cred. Interv.: 0.15; 0.408).

**Table 6:** Intervention efficacy for PWB Autonomy.

		*b*	*s.e.*	*p-*value	*L. L. 95% Cred. Int.*	*U. L. 95% Cred. Int.*
Fixed effects	Constant	4.250	0.252	<0.001	3.766	4.745
	Intervention	−0.056	0.124	0.649	−0.302	0.185
	Time	−0.061	0.055	0.267	−0.172	0.047
	Gender (Female)	−0.122	0.132	0.355	−0.385	0.128
	Age	0.050	0.070	0.475	−0.100	0.183
	Intervention*Time	0.151	0.077	0.049	0.003	0.310
Random effects	L3-Classes: Constant	0.017	0.040		0.001	0.101
	L2-Students: Constant	0.273	0.065		0.150	0.408
	L1-Time: Constant	5.916	2.854		1.825	10.986
	L1-Time: Constant*Time	−0.036	0.029		−0.094	0.019
	L1-Time: Time	−5.475	2.863		−10.575	−1.376

Model Fit D-bar = 617.28; L. L. 95% Cred. Int., Lower Level Bayesian 95% Credible Interval; U. L. 95% Cred. Int., Upper Level Bayesian 95% Credible Interval.

Considering the other five dimensions of the PWB, we found no other significant intervention by time interaction effects while we found a significant effect of the covariate Age on Personal Growth (b = 0.203, SE = 0.077, *p* = 0.009, Bayesian 95% Cred. Interv.: 0.054; 0.358; Supplementary Table S2) and only a significant marginal tendency of time by intervention effect on Purpose in Life (b = 0.13, SE = 0.070, *p* = 0.062, Bayesian 95% Cred. Interv.: −0.005; 0.266; Supplementary Table S3). However, as for the previous outcomes, we found significant school classes and students’ differences for all sub-dimensions of psychological well-being.

Among the five subdimensions of the FFMQ (Table 7; Tables from S7 to S10), a significant fixed intervention by time interaction effect emerged for Acting with awareness (b = 0.197, SE = 0.07, *p* = 0.005, Bayesian 95% Cred. Interv. = 0.058; 0.335, Table 7). On Acting with awareness, we also found a significant effect of intervention (b = 0.300, SE = 0.131, *p* = 0.022, Bayesian 95% Cred. Interv. = 0.044; 0.556) and of time (b = −0.123, SE = 0.049, *p* = 0.012, Bayesian 95% Cred. Interv. = − 0.218; −0.028). Simple slope analysis for Acting with awareness showed a negative and significant linear trend for the Control group (b = −0.12, s.e. = 0.05, p < 0.01), and a positive and non-significant trend for the Intervention group (b = 0.07, s.e. = 0.05, *p* = 0.13). As regards random effects, we found significant variability among students (students L2 σ^2^ = 0.354, SE = 0.067, Bayesian 95% Cred. Interv.: 0.234; 0.496).

**Table 7:** Intervention efficacy for FFMQ Acting with Awareness.

		*b*	*s.e.*	*p-*value	*L. L. 95% Cred. Int.*	*U. L. 95% Cred. Int.*
Fixed effects	Constant	3.588	0.263	0.000	3.074	4.098
	Intervention	0.300	0.131	0.022	0.044	0.556
	Time	−0.123	0.049	0.012	−0.218	−0.028
	Gender (Female)	−0.066	0.138	0.629	−0.335	0.202
	Age	−0.036	0.070	0.605	−0.171	0.104
	Intervention*Time	0.197	0.070	0.005	0.058	0.335
Random effects	L3-Classes: Constant	0.015	0.029		0.001	0.081
	L2-Students: Constant	0.354	0.067		0.234	0.496
	L1-Time: Constant	−0.637	1.702		−3.735	2.443
	L1-Time: Constant*Time	−0.038	0.027		−0.092	0.014
	L1-Time: Time	1.014	1.707		−2.076	4.125

Model Fit D-bar = 551.47; L. L. 95% Cred. Int., Lower Level Bayesian 95% Credible Interval; U. L. 95% Cred. Int., Upper Level Bayesian 95% Credible Interval.

Furthermore, results showed a marginally significant intervention by time interaction effect for Non-reacting (b = −0.137, SE = 0.078, *p* = 0.078, Bayesian 95% Cred. Interv. = − 0.29; 0.017; Supplementary Table S7). Considering simple slope analysis for No-reacting, we found a positive non-significant linear trend for the Control group (b = 0.02, s.e. = 0.05, *p* = 0.75), and a negative and significant effect for the Intervention group (b = −0.12, s.e. = 0.05, *p* = 0.03). Also, for No-Reactivity, we found significant random effects (students L2 σ^2^ = 0.195, SE = 0.059, Bayesian 95% Cred. Interv.: 0.085; 0.315).

Finally, age had a positive effect on the Observing mindfulness facet (b = 0.159, SE = 0.082, *p* = 0.052, Bayesian 95% Cred. Interv. = 0.005; 0.332; Supplementary Table S8) and on the Describing FFMQ facet (b = 0.128, SE = 0.066, *p* = 0.053, Bayesian 95% Cred. Interv. = 0; 0.263; Supplementary Table S9). Indeed, the older the students are, the more they present higher Observing and Describing.

Unlike what was expected, we did not find a significant intervention by time interaction effect on Nature Connectedness (Table 8). The only significant fixed effect, also in this case, was the positive effect of Age (b = 0.111, SE = 0.049, p = 0.022, Bayesian 95% Cred. Interv. = 0.017; 0.208). Moreover, also for Nature Connectedness’s random effects we found significant differences among students (L2 σ^2^ = 0.158, SE = 0.03, Bayesian 95% Cred. Interv.: 0.104; 0.223) and classes (L3 σ^2^ = 0.008, SE = 0.02, Bayesian 95% Cred. Interv.: 0.001; 0.044).

**Table 8:** Intervention efficacy for connectedness to nature.

		*b*	*s.e.*	*p-*value	*L. L. 95% Cred. Int.*	*U. L. 95% Cred. Int.*
Fixed effects	Constant	2.834	0.179	0.000	2.485	3.188
	Intervention	−0.092	0.088	0.298	−0.265	0.083
	Time	0.020	0.032	0.528	−0.043	0.084
	Gender (Female)	0.161	0.094	0.085	−0.024	0.348
	Age	0.111	0.049	0.022	0.017	0.208
	Intervention*Time	0.068	0.046	0.145	−0.023	0.159
Random effects	L3-Classes: Constant	0.008	0.020		0.001	0.044
	L2-Students: Constant	0.158	0.030		0.104	0.223
	L1-Time: Constant	0.928	0.923		−0.328	3.119
	L1-Time: Constant*Time	0.009	0.012		−0.014	0.033
	L1-Time: Time	−0.765	0.926		−2.962	0.494

Model Fit D-bar = 307.75; L. L. 95% Cred. Int., Lower Level Bayesian 95% Credible Interval; U. L. 95% Cred. Int., Upper Level Bayesian 95% Credible Interval.

### Correlations among outcome variables

We calculated correlations between post- and pre-test variations of the main outcomes that resulted significant from multilevel regression model (SHS, PWB Autonomy, Acting with Awareness). Variation in SHS scores correlated with variation in PWB Autonomy scores: the change between pre-test and post-test in subjective happiness was positively correlated with the change between pre-test and post-test in Autonomy (*r* = 0.27*). Moreover, Acting with Awareness variation between pre- and post-test was also positively associated with the variation in Autonomy levels (*r* = 0.20*).

### The effects of age

As we illustrated above, age had a significant role as co-variate in the change of several outcome variables (observing, describing, personal growth, connectedness to nature). To understand the type of association, we performed a bivariate correlation that showed all positive but weak correlations between age and outcome variables at pre-test and post-test. In particular, Age with Personal Growth had a significant positive correlation just at pre-test (*r* = 0.28*), while at post-test, it was positive and non-significant (*r* = 0.12). With the Mindfulness facet of Observing the coefficients were positive and significant both at pre-and post-test (*r* = 0.17*, *r* = 0.18*), while with Describing, the correlation was just significant at pre-test (*r* = 0.17*, *r* = 0.13). Finally, the correlation between Age and Nature Connectedness was positive and significant at pre-(*r* = 0.19*), and post-test (*r* = 0.22**).

### Agreeability and qualitative results on perceived benefits

Descriptive statistics on the two questions on the agreeability, showed that the program was mostly appreciated, scoring higher than the middle point of the scale (M = 3.20; SD = 0.87) on the question “How pleasant was your involvement in the program?” and scoring almost close to the middle point of the scale (M = 2.84; SD = 1.13) on the question “how much were you able to overcome the difficulties encountered during the program?”

Regarding the qualitative analysis, 96 answers were processed through a qualitative content analysis; 29 codes were detected and then following an inductive way of clustering coded data (Schreier, 2012), the codes were grouped into 10 categories and 4 themes following higher level of abstraction (Table 9). The total absolute frequency of codes was 110, indeed 14 answers were codified with two codes, while the remaining 82 answers received just one code.

**Table 9:** Qualitative content analysis of responses on perceived benefits of the program.

Theme	Fr.	Fr.%	Category	Fr.	Fr.%	Code	Fr.	Fr.%
Connection with oneself	48	43.6	Feeling the body	4	3.6	Listening to one’s body messages	3	2.7
						Being more aware of one’s breathing	1	0.9
			Feeling and regulating emotions	8	7.3	Contacting one’s emotions with less fear and inhibition to act	2	1.8
						Better management of emotions	1	0.9
						Better management of anxiety	2	1.8
						Screaming to activate the “warrior” part	1	0.9
						Having fun and laughing with group play	1	0.9
						Overcoming emotional difficulties	1	0.9
			Sense of wellbeing and pleasure	19	17.3	Feeling inner calm	3	2.7
						Feeling a deeper sense of inner wellbeing	5	4.5
						Feeling less stress	6	5.6
						Feeling a sense of relaxation	4	3.6
						Cleaning the mind off from many confused thoughts	1	0.9
			Awareness and personal growth	17	15.5	Raising awareness	6	5.6
						Improving self-understanding	3	2.7
						Developing a deeper connection with oneself	2	1.8
						Having the opportunity to work oneself	2	1.8
						Improving one’s personality	1	0.9
						Enriching knowledge and way of thinking	3	2.7
Connection with others	8	7.3	Emotional looseness to classmates	4	3.6	Feeling emotional closeness to classmates	2	1.8
						Improving listening skills	2	1.8
			Understanding the diversity of others and context	4	3.6	Better understanding of others	3	2.7
						Discussing the influence of school on wellbeing and increasing critical awareness	1	0.9
Connection with nature	31	28.2	Beauty and fascination about Nature	22	20	Feeling in a positive and sensorial touch with nature Feeling a sense of “openness to the world”	18 4	16.5 3.6
			Suffering for the risks and damages to Nature	9	8.2	Being more sensitive to the environmental damages Reflecting on global pollution and climate crisis	7 2	6.4 1.8
Generic answers	23	20.9	Clear generic answers	15	13.6			
			Not clear generic answers	8	7.3			

Most of the participants who identified benefits from the program were grouped in the theme “Connection with oneself” (43.6%). This theme regarded all answers expressing feelings and meanings related to a personal experience of mindfulness and body awareness: body sensations, emotions, awareness and comprehension of one’s own functioning. In this context, the most frequent category was “Experiencing a sense of wellbeing and inner pleasure” followed by “Awareness and personal growth,” “Feeling and regulating emotions,” and finally “Improving body perception and awareness.” In the category “Feeling and regulating emotions,” an example of code was “Overcoming difficulties,” which was used to codify responses such as: “*Talking with my classmates and the psychologist about some topics was important and helpful for me in overcoming my difficulties in emotions*” (Id 92, male, 15 years old) or “Contacting emotions” used to codify answers such as “*I did not know before that there are so many things to emotionally discover and overcome*” (Id 7, male, 15 years old); “O*vercoming some difficulties in managing anxiety*” (Id 16, female, 15 years old).

The second most frequent theme, i.e., “Connection with nature” (28.2%), included a positive component related to the relationship with nature, namely “Beauty and fascination of nature” (20%), which was also the most frequent category among all the categories, and an unpleasant component, referred to the awareness of nature’ suffering because of the damages brought by humans to the other species. Indeed, during the final modules of the program, participants had access to some information on the damage of climate crisis and connected that information with their feelings and personal experiences of climate crisis and ecological transition time. “Climate crisis” category included for instance this code: “Being more sensitive to the environmental damages” that codified answers, such as “*I increased my knowledge on problems concerning the planet and people living on it*” (Id 51, male, 14 years old) and “*I got higher awareness on how my actions affect the environment*” (Id 41, female, 17 years old). An example of a code belonging to the category “Beauty and fascination with nature” was “Contact with nature,” which was used to code responses such as “*Learning to feel the Earth as something closer to me*.” (Id 77, female, 15 years old) or “*To feel an emotional reconnection with the nature that surrounds me*” (Id 24, male, 15 years old).

The third theme in order of frequency brought together answers that refer to a vague concept or a concept not linkable to a precise code, which could be positive (example of answer: “*It was useful for everything*”; Id 26, female, 16 years old) or not (“*I do not know*” Id 66, female, 16 years old).

Finally, the fourth theme concerned the benefits related to “Connecting with others” that encompassed a dimension regarding “emotional closeness” with classmates and a more cognitive dimension of “understanding” the others’ differences and cultural context. It was particularly interesting to underline codes referring to the opportunity to make connections with peers that they did not know before, although they were attending the same class, and to feel less isolated or with a less hostile attitude toward others. An example of the code included in “understanding” category was “Better understanding of others” which was used to codify answers such as “*It helped me to understand better others’ weaknesses*” (Id 33, female, 16 years old) or “*To peacefully talk with others who express different opinions*” (Id 96, male, 15 years old). An example of the code included in “Emotional closeness” category was “Closeness with classmates” used to codify answers such as “It helped to strengthen relationships with classmates” (Id 47, female, 14 years old) or “Feeling closer to my classmates, also the ones I did not know before” (Id 9, male, 15 years old).

## Discussion

The study presented here was aimed at evaluating the effectiveness of a novel mindfulness-based program in improving well-being and ecological self-awareness of adolescent students. The paper focuses on investigating well-being, mindfulness, and connectedness to nature as crucial outcomes of the program, MINDhEARTH, which aimed to bridge the individual (MIND) and socio-ecological spheres of influence (EARTH). Qualitative feedback was also collected from participants regarding the benefits they obtained from the program.

Multilevel regression analysis showed results that partially confirmed the hypotheses. Additional relevant results seem to refer to a variability of effects among students, classes, and age. The first hypothesis was partially confirmed: the program affected both hedonic and eudaimonic well-being. A key result was the increase in subjective happiness within the experimental group. Participants reported feeling happier at the end of the program, consistent with other studies on mindfulness interventions that have shown increases in outcomes related to quality of life (Anand et al., 2021), subjective well-being (Primasari and Yuniarti, 2021), and prevalence of positive emotions (Tang et al., 2019). However, the present findings regarding subjective well-being contrast with other research that showed mindfulness to affect various dimensions of psychological well-being but not positive affect and life satisfaction (Scafuto et al., 2023). The result could be due to the difference between this program and the previous ones (Scafuto et al., 2023), and the combination of mindfulness with tools of dialog and group facilitation, playful games and nature-based activities that may have increased the agreeability and an immediate sense of pleasure and positive affect. Agreeability, indeed, has been recognized as a critical point of MBIs in adolescents (Kuyken et al., 2022) that should be seriously considered since no program can work if it does not meet needs, motivation, and effective participation of the youth. MINDhEARTH was ideated as a program but it expressed flexibility to adapt to the needs of the classes and encountered in average a good level of agreeability (higher than the middle point of the scale), while participants also felt like overcoming initial difficulties encountered for what they perceived as a completely novel and not familiar experience.

Among the dimensions of psychological wellbeing, autonomy was enhanced by the MINDhEARTH program. Bivariate correlations between post- and pre-test variations in autonomy scores and the remaining significant outcome variations, showed that autonomy was correlated with both subjective happiness and acting with awareness. In other words, the variation in eudaimonic well-being (i.e., autonomy), as a result of participation in the MINDhEARTH program, was positively and significantly correlated with the variation in hedonic wellbeing and mindfulness (i.e., acting with awareness).

Previous studies on the correspondence between some dimensions of basic human values and psychological well-being in adolescents emphasized a key role that autonomy may play in promoting social and environmental change. For instance, a study revealed how autonomy was positively correlated with openness to change and negatively correlated with conservation values (Bojanowska and Piotrowski, 2018). The result of a main effect on autonomy seems in line with the hypothesis that mindfulness would primarily foster conscious personal choices toward a consistency between values and behaviors. According to Self-Determination Theory (Brown and Ryan, 2003), mindfulness would lead to a lifestyle perceived as self-congruent, enduring overtime, and aligned with one’s core values. In a still unsustainable society, where prevalent consumeristic habits and a capitalistic view of exploitation damage nature and biodiversity, it seems necessary to adopt a non-conformist stance and critical thinking - thus fostering a dimension of independence and self-regulation (autonomy) - to promote social change, starting from one’s own lifestyle. However, the present results on autonomy should be interpreted with caution due to the result being marginally significant (positive but not significant trend in the experimental group) and the low reliability of the measure in the present sample.

Regarding the other dimensions of psychological well-being, we also found a marginally significant interaction effect between intervention and time on purpose in life, suggesting that the program also marginally contributed at increasing adolescents’ feelings and beliefs that life has a meaning and that what happens is only a part of a bigger picture, in line with previous studies (Scafuto et al., 2024a). This result recalls the Mindfulness-to-Meaning Theory (Garland et al., 2015), which states that mindfulness fosters metacognitive skills, thanks to meaning-making and an enlargement of attentional scope, that allows to notice new information useful for reviewing interpretation of events. Feeling that every event, even though unpleasant, can be meaningfully framed in a bigger picture that could be not fully understood but has coherence and meaning, is a relevant eudaimonic aspect of well-being that helps to cultivate hope and trust in future, especially needed in times of uncertainty. Indeed, the program was inspired by the Work that reconnects, also named the authors “Active hope” since it is aimed to actively build a new social narrative up, alternative to catastrophism and denialism (Macy and Brown, 2014).

A significant interactive effect between intervention and time did not appear on personal growth, differently from what was expected, even though students reported codes in qualitative answers that can recall this dimension of well-being. Differently, a significant main effect of the covariate age was found, indicating that the older the students were, the more they expressed personal growth or a positive attitude toward novel experiences. This was in line with similar previous research in adolescents (Viejo et al., 2018), which observed a developmental trend where older adolescents reported higher scores on the life development scale, a construct closely related to personal growth. Age also affected the mindful facets of observing and describing, in line with recent literature showing that mindfulness facets are positively related to age (Mahlo and Windsor, 2020) and that age may moderate the effects of mindfulness-based interventions on psychological distress (Gómez-Odriozola and Calvete, 2021). These findings may be attributed to the progressive development of metacognitive abilities with age (Weil et al., 2013), which enhances individuals’ capacity to observe thoughts and emotions with greater awareness and less reactivity.

Examining the study hypothesis regarding the influence of the MINDhEARTH program on well-being through the qualitative analysis of participants’ reports on perceived program benefits, it emerged that the category of well-being was the most frequently mentioned (cfr. “Sense of well-being and pleasure”). The finding that participants reported an enhanced sense of well-being through engagement in the program aligns with prior observations from studies using other mindfulness interventions for children and adolescents, such as the junior-Mindfulness Oriented Meditation program (Matiz et al., 2024), or the MindUP program (Maloney et al., 2016). Regarding the two components of well-being, participants’ responses focused more on the hedonic rather than eudaimonic component of well-being, with perceived outcomes such as “inner calm,” “inner well-being,” “less stress,” and “a sense of relaxation.” Moreover, another category of responses, that of “Awareness and personal growth” can be linked to enhanced well-being, and identifies the availability of developing one’s capabilities, gaining new skills, and enhancing understanding of oneself through every experience of life.

Regarding the second study hypothesis (i.e., effectiveness of the program in improving mindfulness skills), it was found that programs significantly affected the mindful facet of acting with awareness, but not the mindful facet of observing, as expected. Slope analyses showed that acting with awareness significantly decreased in the control group, while it did not significantly increase in the experimental group. The non-significant but positive trend observed in the intervention group could be explained by the possibility that achieving a significant increase of acting with awareness may require a longer duration of practice than the 18 h provided by the MINDhEARTH program as well as a longer follow-up period to observe the intervention’s effects over time. Indeed, some studies identify acting with awareness as a secondary outcome to be achieved, whose increases are more likely observed in people who have a longer and regular practice of mindfulness (Hunecke and Richter, 2019).

The impact of the program on mindfulness skills reported through the qualitative analysis on perceived benefits, showed that the theme “Connection with oneself,” that is linked to mindfulness skills, was the most frequent and it included the categories named: “Feeling the body,” “Feeling and regulating emotions,” and “Awareness and personal growth.” Recalling Hölzel et al. (2011), adolescents reported some main mechanisms through which MBIs would produce positive effects on well-being, that are similar to these categories: body awareness, emotion regulation, the change in the perspective on the self, that could be seen like the category “Awareness and personal growth.”

Furthermore, among the other facets of mindfulness, an interesting result emerged from the analysis of non-reacting. A significant negative effect was found in the intervention group, while a positive but not significant trend was observed in the control group. This result contrasts with previous literature, which found increases in non-reacting following mindfulness-based programs (Benzo et al., 2018). One possible explanation for this discrepancy relates to the specific design of the MINDhEARTH program. Indeed, some practices within the program focused on sympathetic system activation, while others, especially in the final modules, emphasized action/problem-oriented coping strategies for facing climate stressors. Even qualitative feedback from participants highlighted benefits such as stimulating one’s agency and recognizing one’s emotions with less fear and inhibition (see the category “Feeling and regulating emotions”). The activation of a self-defense stance may also increase reactivity and could be viewed as a positive transition in the emotion regulation processes, shifting from a not-intentional inhibition of action to intentional disinhibition (see Kunz, 2014). This result might be particularly relevant, considering the higher prevalence of internalizing problems (i.e., withdrawal, inaction, anxiety, depression) among adolescents in the Italian context compared to externalizing problems (Frigerio et al., 2009), as well as the global increase in internalizing problems after the Covid-19 Pandemic (WHO & UNICEF, 2023).

Finally, the third hypothesis of a significant change in connectedness to nature was partially disconfirmed. The only significant fixed effect - probably explained as well by increasing metacognitive skills - was once again linked to the covariate of age: the older the students were, the more they felt connected to nature. The absence of a significant interaction between intervention and time for nature connectedness may be related to the baseline difference between the two participant groups (with controls scoring higher than MINDhEARTH participants).

Nevertheless, by integrating quantitative data with qualitative reports, we found that connectedness to nature was the second most frequently cited theme, after connectedness to oneself. Participants reported feeling a more positive sensorial and emotional link to nature, aligning with the concept of nature connectedness and affiliation (Barbiero and Berto, 2016). They also expressed a sense of expanding their perspective to the world, consistent with the concept of widening (Macy and Brown, 2014), and reported feeling more sensitive toward the suffering of nature. Regarding the apparent inconsistency between results on connectedness with nature obtained through quantitative and qualitative methods, an explanation could be due to the difference between the two samples: qualitative sample included outliers that were removed for statistical reasons from the quantitative analysis.

In sum, MINDhEARTH program partially affected connectedness to nature that increased in the intervention group, compared to the control, but not significantly, and was reported as the second main benefit in the qualitative reports. This finding is in line with literature that considers connectedness to nature as a stable trait-like variable, even though affected by experiences such as visiting greenspaces around the house or influences from friends, families and educational contexts (Oh et al., 2021). CN identifies the feeling of emotional bond and identification with nature, the preference of experiences in nature even if bearing personal costs, such as practical discomfort, and can be seen as a psychological indicator of the main construct, biophilia, that is an innate tendency to manifest attention and affiliation for living systems, but it would need experience and a proper educational context to fully develop (Barbiero and Berto, 2016). In a society that is characterized by alienation from nature (Chalquist, 2009), the finding of just a partial and not significant impact on CN is not surprising, especially if we consider school constraints to deliver the outdoor activities of the program and the low dose and frequency that was allowed (18 h).

However, mindfulness combined with connectedness to nature are key factors that improve the experience in nature and can increase the benefits for the stress recovery (Ulrich et al., 1991) and for attention restoration in nature (Kaplan, 1995). Mindfulness programs, such as MINDhEARTH, may influence how an individual perceives nature and relates with it and, this, in turn, affects how a person can benefit from visiting nature. Indeed, mindfulness was found to increase relatedness to nature (Scafuto, 2019) while connectedness to nature was found to modulate the influence of nature exposure on well-being measures (Chang et al., 2024). People who are more mindfully connected with nature can benefit more from nature exposure because they tend to be more conscious and mindful of the nature around them and more attentive, for instance, to biodiversity (Fuller et al., 2007). In summary, they would actively and intentionally interact with nature, not only frequently visiting greenspace.

In addition to fixed effects, it is also worth discussing the random effects, which were found to be significant for all the outcome variables. Random effects were included to account for the variability in outcomes and highlighted the need for future program adjustments targeting specific groups to better control this variability within the research design. Indeed, the observed variability in program effects among students may be due to pre-existing differences both within and between classes, such as diverse learning abilities and difficulties, as well as varying cultural and ethnic backgrounds.

Furthermore, the class climate, that is affected particularly by the consistency among school norms, perceived social support and sense of community (Sigmon et al., 2002) is a key variable that we did not control for, but which could have influenced the different responses observed across classes to the delivered program. Some classes exhibited a collaborative emotional climate, whereas others exhibited a more oppositional attitude toward adults and school in general. These latter classes lacked social cohesion, and their attention appeared to be more dependent on external reinforcements such as school grades or sanctions. In such classes, it was harder to establish an active and cooperative climate, suggesting that they could benefit from a longer intervention that involves teachers’ active participation and norms’ consistency.

The idea that the contextual constraints of the class climate and school system may negatively affect individual well-being and related psychological outcomes, or at least fail to produce positive effects, can be supported by the observed decline in acting with awareness and negative trends in well-being measures (even though non-significant) within the control group, which did not participate in the MINDhEARTH program. These results are in line with previous studies by the authors, which showed a deterioration over time in internalizing and externalizing problems (Scafuto et al., 2022), psychological well-being (Scafuto et al., 2023) and self-enhancement values (Scafuto et al., 2025) among control participants.

### Strengths, limitations, and implications for future research

This program had the innovative value to combine mindfulness with connectedness to nature practices and to study its benefits through quantitative and qualitative measures, together with its agreeability in youth. To our knowledge, this is the first attempt at applying nature-based mindfulness programs in adolescents. Most participants recognized benefits in the three areas at the basis of the program’s ideation: a sense of connectedness to oneself, others, and nature. In the era of a virtually hyper-connected society where we paradoxically assist to a widespread perception of isolation with the implication on mental health problems in adolescents (Narváez Carrión et al., 2024), designing interventions aimed to foster a sense of interconnectedness promotes individual wellbeing and may promote relational and social wellbeing as well.

In addition, the program proved to be feasible since didactic contents and aims of the program were in line with the school’s requirements for both emotional education (for the first four modules) and civic education (for the last two modules), getting the approval of the teachers’ team and the principals. This correspondence could make the program part of the general curriculum of the public high schools, also considering the relatively brief time required (18 h).

Nevertheless, some limitations should be underlined regarding the methods and the contextual constraints to the intervention. First, method limitations include the not randomized type of design that was not feasible in these schools, the absence of active control, the sample size that did not allow further investigation on interactive effects for instance of time, intervention, and age. In addition, further measures should be considered, especially regarding hedonic well-being, which was the study’s main result. Indeed, subjective wellbeing was measured only with subjective happiness scale. Although subjective wellbeing is generally considered to coincide with subjective happiness, some studies have shown that there is just a partial overlap between the two constructs (Lyubomirsky and Lepper, 1999). A relevant limitation is also the low reliability coefficient of the autonomy subscale of the Psychological Well-Being that suggests being cautious the interpret the results of a positive effect of the program on autonomy. Another limitation derived from the shortness of the qualitative answers, may be explained since the question about what type of perceived benefits from the program was asked as a single ending question of the quantitative questionnaire, pointing out for future studies the call for interviews for more extended qualitative analysis.

In addition, a comment is needed on our choice to exclude and not to “normalize” multivariate outliers to keep the intervention effect unaffected. We would stress here, again, that the decision to exclude the multivariate outliers was only determined by the fact that assumptions of the models we used to test our hypotheses require normal data. The exclusion of outliers does not entail an automatic “disqualification” of participants who provided the outlier, but due to the uncertainty of the reasons students provided outlier responses, our safest choice was not to consider data coming from outliers, rather than transforming the data to normality and including them in the analysis. The decision to analyze only normally distributed data allowed us to trust and comment on our results according to the literature without thinking that normality transformations biased our reasoning. Differently, adding transformed-to-normality data to the rest of the data would have shed doubts that our results and conclusions were partly or totally due to this decision. These doubts are supported by the fact that the transformations for normality are irrespective of the different sources of systematic variances acting on the two comparison groups (the effect of intervention and the absence of the impact of the intervention). These unpredictable consequences of normality transformation would have left the doubt that our results were due to this decision, and that our discussion was consequently biased. To conclude, we consider our choice was the safest for maintaining the analysis process as clean and clear as possible.

Limitations regarding the intervention are related to the school context constraints. For instance, the classrooms were not large enough to allow plenty of movement for the participants during the sessions that required larger body movements. As suggested by Baelen et al. (2023), structural characteristics of classrooms—such as their physical or organizational setup—may represent a critical factor influencing both the implementation and the effectiveness of school-based mindfulness programs. School environment is, indeed, considered one of the main implementation variables to be considered along with teacher-related skills, training, and program characteristics (Lo, 2024). The organization of the spaces reflects the ongoing traditional frontal teaching of the Italian school system. The proposal of any interactive way of teaching, that especially involves body movement and activities in nature, still encounters numerous difficulties that need to be addressed in future programs to improve effectiveness especially on connectedness to nature variables.

A second limitation about the delivery of the intervention relates to the fact that only external instructors led the program while in the future trained teachers in MINDhEARTH could be empowered and directly deliver the program through specific training, personal practice, and ongoing supervision, to ensure the effectiveness of the intervention (Baelen et al., 2023) and achieving the general aim of promoting well-being and reinforcing connectedness to nature in all of the educational community. Indeed, individuals can be encouraged to adopt ecological behaviors and not feel helpless in respect to huge global challenges such as climate change, if they are supported by a larger community, if they identify with a community of activists, and thus, if mindful actions become more collective and widespread in the organization they belong to, becoming norms to comply with (Scafuto and La Barbera, 2016).

Although mindfulness-based interventions have shown efficacy in populations with ADHD and ASD (Leeth et al., 2019), a third limitation concerns the lack of adaptation of the program to the specific needs of these students, who received the same intervention as their neurotypical peers. Having numerous and heterogeneous classes in small rooms was not a favorable factor for allowing a mindfulness-based climate. External disruptive factors (such as interruptions from hyperactive and non-attentive students) were not facilitating the flow of the activities especially in the first sessions of the program, when the relationship with the expert was still being built.

A fourth limitation is the lack of socio-demographic information about participants (e.g., socioeconomic status) and individual data on special needs or learning disorders or disabilities, which could influence program outcomes and interpretation of the results. Future research should negotiate with teachers‘committee to collect more sensitive information on individual level, establishing a more trustful relationship between researchers and the school community.

Future adaptation of the program could incorporate targeted techniques and competencies designed to address neurodiverse profiles (Leeth et al., 2019), thereby enhancing its effectiveness across all participant groups. In this regard, mindfulness-based interventions (MBIs) in educational settings aimed at the inclusion of students with disabilities may particularly benefit from the adaptation of core mindfulness techniques—such as focused breathing, body awareness, and present-moment attention—to meet the specific cognitive and sensory needs of these students (Bello et al., 2023). Such adaptations may also extend to the learning environment itself, including the presence of additional instructors, preparatory meetings with support teachers, access to open and flexible spaces, and the integration of more breaks within longer sessions (Clemson and Coyle, 2025).

Some suggestions about future interventions can be drawn from the current findings and limitations. Further studies would benefit from longer intervention integrated with daily practice and evaluation timeframes with continuing meditation practice support, especially considering that connectedness to nature and ecological behavior may be in a more distal relationship with mindfulness. Indeed, the dose and the frequency of mindfulness practices are relevant moderator factors in the maintenance of results in adolescents (Volanen et al., 2020).

Finally, given the relevance of age as co-variate factor, next programs could be addressed to older adolescents because of their neurocognitive maturity, plasticity to change, and metacognitive skills (Kuyken et al., 2022), while tailoring new programs that involve, for instance, more ecopsychology activities and green mindfulness for younger adolescents. For instance, the development of MINDhEARTH could expand the final modules of MindAction, with a co-designing between the facilitators and the participants of outdoor and indoor spaces that promote wellbeing. These modules could reinforce motivation, sense of agency, and accomplish the need for socialization in first-year high school students.

The program hereby presented is an attempt to integrate mindfulness with nature-based practices to involve adolescents and increase well-being. The crucial key for future implementation is to enhance awareness of one health paradigm, whereby personal well-being is strongly connected to planetary well-being and collective action is driven by the perception of our common destiny as terrestrial, a sense of interdependence with other living species, and a development of a more attentive and mindful way to connect with nature, reinforcing biophilic attitudes.

## Data Availability

The raw data supporting the conclusions of this article will be made available by the authors, without undue reservation.

## References

[ref1] Amel E. L. Manning C. M. Scott B. A. (2009). Mindfulness and sustainable behavior: pondering attention and awareness as means for increasing green behavior. Ecopsychology 1, 14–25. doi: 10.1089/eco.2008.0005

[ref2] American Psychological Association (2020). Stress in America™ 2020: A National Mental Health Crisis. Washington: APA.

[ref3] Anand P. Bakhshi A. Gupta R. Mridula B. (2021). Gratitude and quality of life among adolescents: the mediating role of mindfulness. Trends Psychol 29, 706–718. doi: 10.1007/s43076-021-00077-z

[ref9001] Baceviciene M. Jankauskiene R. (2022). The mediating effect of nature restorativeness, stress level, and nature connectedness in the association between nature exposure and quality of life. International Journal of Environmental Research and Public Health, 19:2098. doi: 10.3390/ijerph19042098 35206285 PMC8871825

[ref5] Baelen R. N. Gould L. F. Felver J. C. Schussler D. L. Greenberg M. T. (2023). Implementation reporting recommendations for school-based mindfulness programs. Mindfulness 14, 255–278. doi: 10.1007/s12671-022-01997-2

[ref6] Baer R. A. Carmody J. Hunsinger M. (2012). Weekly change in mindfulness and perceived stress in a mindfulness-based stress reduction program. J. Clin. Psychol. 68, 755–765. doi: 10.1002/jclp.21865, PMID: 22623334

[ref7] Baer R. A. Smith G. T. Hopkins J. Krietemeyer J. Toney L. (2006). Using self-report assessment methods to explore facets of mindfulness. Assessment 13, 27–45. doi: 10.1177/1073191105283504, PMID: 16443717

[ref8] Barbaro N. Pickett S. M. (2016). Mindfully green: examining the effect of connectedness to nature on the relationship between mindfulness and engagement in pro-environmental behavior. Personal. Individ. Differ. 93, 137–142. doi: 10.1016/j.paid.2015.05.026

[ref9] Barbiero G. Berto R. (2016). Introduzione alla Biofilia. La relazione con la Natura tra Genetica e Psicologia. Roma: Carocci Editore.

[ref10] Barrett B. Grabow M. Middlecamp C. Mooney M. Checovich M. M. Converse A. K.. (2016). Mindful climate action: health and environmental co-benefits from mindfulness-based behavioral training. Sustainability 8:1040. doi: 10.3390/su8101040, PMID: 28008371 PMC5170843

[ref11] Bello C. M. Blagrave A. J. Lytle R. K. (2023). Keys to success: mindfulness-based practices in the classroom for stress and anxiety in students with disabilities. J. Phys. Educ. Recreat. Dance 94, 29–38. doi: 10.1080/07303084.2023.2252850

[ref12] Benzo R. P. Anderson P. M. Bronars C. Clark M. (2018). Mindfulness for healthcare providers: the role of non-reactivity in reducing stress. Explore 14, 453–456. doi: 10.1016/j.explore.2018.03.008, PMID: 30292600 PMC6295257

[ref13] Bojanowska A. Kaczmarek Ł. D. (2022). How healthy and unhealthy values predict hedonic and Eudaimonic wellbeing: dissecting value-related beliefs and Behaviours. J. Happiness Stud. 23, 211–231. doi: 10.1007/s10902-021-00396-z PMC9530432 36213306

[ref14] Bojanowska A. Piotrowski K. (2018). Values and psychological well-being among adolescents – are some values ‘healthier’ than others? Eur. J. Dev. Psychol. 16, 402–416. doi: 10.1080/17405629.2018.1438257

[ref15] Brown K. W. Kasser T. (2005). Are psychological and ecological wellbeing compatible? The role of values, mindfulness, and lifestyle. Soc. Indic. Res. 74, 349–368. doi: 10.1007/s11205-004-8207-8

[ref16] Brown K. W. Ryan R. M. (2003). The benefits of being present: mindfulness and its role in psychological wellbeing. J. Pers. Soc. Psychol. 84, 822–848. doi: 10.1037/0022-3514.84.4.822, PMID: 12703651

[ref17] Capaldi C. A. Dopko R. L. Zelenski J. M. (2014). The relationship between nature connectedness and happiness: a meta-analysis. Front. Psychol. 5:976. doi: 10.3389/fpsyg.2014.00976, PMID: 25249992 PMC4157607

[ref18] Carsley D. Khoury B. Heath N. L. (2018). Effectiveness of mindfulness interventions for mental health in schools: a comprehensive meta-analysis. Mindfulness 9, 693–707. doi: 10.1007/s12671-017-0839-2

[ref19] Castelpietra G. Knudsen A. K. S. Agardh E. E. Armocida B. Beghi M. Iburg K. M.. (2022). The burden of mental disorders, substance use disorders and self-harm among young people in Europe, 1990-2019: findings from the global burden of disease study 2019. Lancet Regional Health 16:100341. doi: 10.1016/j.lanepe.2022.100341, PMID: 35392452 PMC8980870

[ref20] Chang C. Lin B. B. Feng X. Andersson E. Gardner J. Astell-Burt T. (2024). A lower connection to nature is related to lower mental health benefits from nature contact. Sci. Rep. 14:6705. doi: 10.1038/s41598-024-56968-5, PMID: 38509180 PMC10954714

[ref9002] Chalquist C. (2009). A look at the ecotherapy research evidence. Ecopsychology, 1, 64–74. doi: 10.1089/eco.2009.0003

[ref21] Choe E. Y. Jorgensen A. Sheffield D. (2020). Does a natural environment enhance the effectiveness of mindfulness-based stress reduction (MBSR)? Examining the mental health and wellbeing, and nature connectedness benefits. Landsc. Urban Plan. 202:103886. doi: 10.1016/j.landurbplan.2020.103886

[ref22] Ciacchini R. Orrù G. Cucurnia E. Sabbatini S. Scafuto F. Lazzarelli A.. (2023). Social media in adolescents: a retrospective correlational study on addiction. Children 10:278. doi: 10.3390/children10020278, PMID: 36832407 PMC9954802

[ref23] Cilar L. Štiglic G. Kmetec S. Barr O. Pajnkihar M. (2020). Effectiveness of school-based mental well-being interventions among adolescents: a systematic review. J. Adv. Nurs. 76, 2023–2045. doi: 10.1111/jan.14408, PMID: 32363607

[ref24] Clemson H. G. Coyle D. (2025). Space matters: creating inclusive learning spaces for pupils with additional support needs (ASN) in Scotland. Learn. Environ. Res. 28, 65–79. doi: 10.1007/s10984-025-09526-3

[ref25] Compas B. E. Jaser S. S. Bettis A. H. Watson K. H. Gruhn M. A. Dunbar J. P.. (2017). Coping, emotion regulation, and psychopathology in childhood and adolescence: a meta-analysis and narrative review. Psychol. Bull. 143, 939–991. doi: 10.1037/bul0000110, PMID: 28616996 PMC7310319

[ref26] Costello A. Abbas M. Allen A. Ball S. Bell S. Bellamy R.. (2009). Managing the health effects of climate change: lancet and University College London Institute for Global Health Commission. Lancet (London, England) 373, 1693–1733. doi: 10.1016/S0140-6736(09)60935-1, PMID: 19447250

[ref27] Crescentini C. Capurso V. Furlan S. Fabbro F. (2016). Mindfulness-oriented meditation for primary school children: effects on attention and psychological well-being. Front. Psychol. 7:805. doi: 10.3389/fpsyg.2016.00805, PMID: 27375510 PMC4894866

[ref28] Danon M. (2020). Ecopsicologia. Come sviluppare una nuova consapevolezza ecologica. Roma: Feltrinelli.

[ref29] Di Fabio A. Bucci O. (2016). Green positive guidance and green positive life counseling for decent work and decent lives: some empirical results. Front. Psychol. 7:261. doi: 10.3389/fpsyg.2016.00261, PMID: 26973563 PMC4773606

[ref30] Di Giuseppe M. Ciacchini R. Piarulli A. Nepa G. Conversano C. (2019). Mindfulness dispositions and defense style as positive responses to psychological distress in oncology professionals. Eur. J. Oncol. Nurs. 40, 104–110. doi: 10.1016/j.ejon.2019.04.003, PMID: 31229199

[ref31] Diener E. (2000). Subjective wellbeing: the science of happiness and a proposal for a national index. Am. Psychol. 55, 34–43. doi: 10.1037/0003-066X.55.1.34, PMID: 11392863

[ref32] Dunning D. Tudor K. Radley L. Dalrymple N. Funk J. Vainre M.. (2022). Do mindfulness-based programmes improve the cognitive skills, behaviour and mental health of children and adolescents? An updated meta-analysis of randomised controlled trials. Evid. Based Ment. Health 25, 135–142. doi: 10.1136/ebmental-2022-300464, PMID: 35820989 PMC9340039

[ref33] Ericson T. Kjønstad B. G. Barstad A. (2014). Mindfulness and sustainability. Ecol. Econ. 104, 73–79. doi: 10.1016/j.ecolecon.2014.04.007

[ref34] Feruglio S. Pascut S. Matiz A. Paschetto A. Crescentini C. (2022). Effects of mind-body interventions on adolescents’ cooperativeness and emotional symptoms. Behav. Sci. 12:33. doi: 10.3390/bs12020033, PMID: 35200284 PMC8869189

[ref35] Frigerio A. Rucci P. Goodman R. Ammaniti M. Carlet O. Cavolina P.. (2009). Prevalence and correlates of mental disorders among adolescents in Italy: the PrISMA study. Eur. Child Adolesc. Psychiatry 18, 217–226. doi: 10.1007/s00787-008-0720-x, PMID: 19165539

[ref36] Fuller R. A. Irvine K. N. Devine-Wright P. Warren P. H. Gaston K. J. (2007). Psychological benefits of greenspace increase with biodiversity. Biol. Lett. 3, 390–394. doi: 10.1098/rsbl.2007.0149, PMID: 17504734 PMC2390667

[ref37] Garland E. L. Farb N. A. Goldin P. R. Fredrickson B. L. (2015). The mindfulness-to-meaning theory: extensions, applications, and challenges at the attention–appraisal–emotion interface. Psychol. Inq. 26, 377–387. doi: 10.1080/1047840X.2015.1092493

[ref9003] Ghiroldi S. Scafuto F. Montecucco N. F. Presaghi F. Iani L. (2020). Effectiveness of a school-based mindfulness intervention on children’s internalizing and externalizing problems: The Gaia project. Mindfulness, 11, 2589–2603. doi: 10.1007/s12671-020-01473-9

[ref38] Giovannini C. Giromini L. Bonalume L. Tagini A. Lang M. Amadei G. (2014). The Italian five facet mindfulness questionnaire: a contribution to its validity and reliability. J. Psychopathol. Behav. Assess. 36, 415–423. doi: 10.1007/s10862-013-9403-0

[ref39] Gómez-Odriozola J. Calvete E. (2021). Effects of a mindfulness-based intervention on adolescents’ depression and self-concept: the moderating role of age. J. Child Fam. Stud. 30, 1501–1515. doi: 10.1007/s10826-021-01953-z

[ref40] Grabow M. Bryan T. Checovich M. M. Converse A. K. Middlecamp C. Mooney M.. (2018). Mindfulness and climate change action: a feasibility study. Sustainability 10:1508. doi: 10.3390/su10051508, PMID: 31588364 PMC6778663

[ref41] Haller H. Breilmann P. Schröter M. Dobos G. Cramer H. (2021). A systematic review and meta-analysis of acceptance-and mindfulness-based interventions for DSM-5 anxiety disorders. Sci. Rep. 11:20385. doi: 10.1038/s41598-021-99882-w, PMID: 34650179 PMC8516851

[ref42] Hartig T. Mang M. Evans G. W. (1991). Restorative effects of natural environment experiences. Environ. Behav. 23, 3–26.

[ref43] Herbst M. (2024). The state of the world's children 2024: the future of childhood in a changing world. United Nations children’s fund. Available online at: https://www.unicef.org/reports/state-of-worlds-children-2024 (Accessed April 3, 2025).

[ref9004] Hölzel B. K. Lazar S. W. Gard T. Schuman-Olivier Z. Vago D. R. Ott U. (2011). How does mindfulness meditation work? Proposing mechanisms of action from a conceptual and neural perspective. Perspectives on psychological science: a journal of the Association for Psychological Science, 6, 537–559. doi: 10.1177/1745691611419671 26168376

[ref44] Howell A. J. Dopko R. L. Passmore H. A. Buro K. (2011). Nature connectedness: associations with wellbeing and mindfulness. Personal. Individ. Differ. 51, 166–171. doi: 10.1016/j.paid.2011.03.037

[ref45] Hunecke M. Richter N. (2019). Mindfulness, construction of meaning, and sustainable food consumption. Mindfulness 10, 446–458. doi: 10.1007/s12671-018-0986-0

[ref47] Iani L. Lauriola M. Layous K. Sirigatti S. (2013). Happiness in Italy: translation, factorial structure and norming of the subjective happiness scale in a large community sample. Soc. Indic. Res. 118, 1007–1022. doi: 10.1007/s11205-013-0468-7

[ref49] Jacob J. Jovic E. Brinkerhoff M. B. (2009). Personal and planetary wellbeing: mindfulness meditation, pro-environmental behavior and personal quality of life in a survey from the social justice and ecological sustainability movement. Soc. Indic. Res. 93, 275–294. doi: 10.1007/s11205-008-9308-6

[ref50] Johnson C. Burke C. Brinkman S. Wade T. (2017). A randomized controlled evaluation of a secondary school mindfulness program for early adolescents: do we have the recipe right yet? Behav. Res. Ther. 99, 37–46. doi: 10.1016/j.brat.2017.09.001, PMID: 28910673

[ref51] Kabat-Zinn J. (2003). Mindfulness-based interventions in context: past, present, and future. Clin. Psychol. Sci. Pract. 10, 144–156. doi: 10.1093/clipsy.bpg016, PMID: 40800121

[ref52] Kang Y. Rahrig H. Eichel K. Niles H. F. Rocha T. Lepp N. E.. (2018). Gender differences in response to a school-based mindfulness training intervention for early adolescents. J. Sch. Psychol. 68, 163–176. doi: 10.1016/j.jsp.2018.03.004, PMID: 29861026 PMC6174072

[ref53] Kaplan S. (1995). The restorative benefits of nature: toward an integrative framework. J. Environ. Psychol. 15, 169–182.

[ref54] Khoury B. Sharma M. Rush S. E. Fournier C. (2015). Mindfulness-based stress reduction for healthy individuals: a meta-analysis. J. Psychosom. Res. 78, 519–528. doi: 10.1016/j.jpsychores.2015.03.009, PMID: 25818837

[ref1000] Kleinman K. Sakrejda A. Moyer J. Nugent J. Reich N. Obeng D. (2021). clusterPower: Power calculations for cluster-randomized and cluster-randomized crossover trials, 2021. R package version 0.7. 0. Available online at: https://CRAN.R-project.org/package=clusterPower (Accessed May 4, 2025)., PMID: 25818837

[ref55] Kraines M. A. Peterson S. K. Tremont G. N. Beard C. Brewer J. A. Uebelacker L. A. (2022). Mindfulness-based stress reduction and mindfulness-based cognitive therapy for depression: a systematic review of cognitive outcomes. Mindfulness 13, 1126–1135. doi: 10.1007/s12671-022-01841-7, PMID: 36059888 PMC9436005

[ref56] Kunz E. (2014). Henri Laborit and the inhibition of action. Dialogues Clin. Neurosci. 16, 113–117. doi: 10.31887/DCNS.2014.16.1/ekunz, PMID: 24733976 PMC3984888

[ref57] Kuyken W. Ball S. Crane C. Ganguli P. Jones B. Montero-Marin J.. (2022). Effectiveness and cost-effectiveness of universal school-based mindfulness training compared with normal school provision in reducing risk of mental health problems and promoting well-being in adolescence: the MYRIAD cluster randomised controlled trial. Evidence Based Mental Health 25, 99–109. doi: 10.1136/ebmental-2021-300396, PMID: 35820992 PMC9340028

[ref58] Landry N. Gifford R. Milfont T. L. Weeks A. Arnocky S. (2018). Learned helplessness moderates the relationship between environmental concern and behavior. J. Environ. Psychol. 55, 18–22. doi: 10.1016/j.jenvp.2017.12.003

[ref59] Lassander M. Hintsanen M. Suominen S. Mullola S. Vahlberg T. Volanen S. M. (2021). Effects of school-based mindfulness intervention on health-related quality of life: moderating effect of gender, grade, and independent practice in cluster randomized controlled trial. Qual. Life Res. Int. J. Qual. Life Asp. Treat. Care Rehab. 30, 3407–3419. doi: 10.1007/s11136-021-02868-4, PMID: 34169412 PMC8602227

[ref60] Leeth C. D. Villarreal V. Styck K. M. (2019). Mindfulness interventions for children and adolescents with ADHD: a review of objectives and skills. J. Creat. Ment. Health 14, 436–446. doi: 10.1080/15401383.2019.1625840

[ref61] Lo H. H. M. (2024). Mindfulness for children, adolescents, and families: Integrating research into practice. Cham, Switzerland: Springer.

[ref62] Loy L. S. Reese G. (2019). Hype and hope? Mind-body practice predicts pro-environmental engagement through global identity. J. Environ. Psychol. 66:101340. doi: 10.1016/j.jenvp.2019.101340

[ref63] Lymeus F. Lindberg P. Hartig T. (2018). Building mindfulness bottom-up: meditation in natural settings supports open monitoring and attention restoration. Conscious. Cogn. 59, 40–56. doi: 10.1016/j.concog.2018.01.008, PMID: 29438869

[ref64] Lyubomirsky S. Lepper H. S. (1999). A measure of subjective happiness: preliminary reliability and construct validation. Soc. Indic. Res. 46, 137–155.

[ref65] Macy J. Brown M. (2014). Coming back to life: The updated guide to the work that reconnects. Gabriola, Canada: New Society Publishers.

[ref66] Mahlo L. Windsor T. D. (2020). Older and more mindful? Age differences in mindfulness components and well-being. Aging Ment. Health 25, 1320–1331. doi: 10.1080/13607863.2020.1734915, PMID: 32114803

[ref9005] Maloney J. E. Lawlor M. S. Schonert-Reichl K. A. Whitehead J. (2016). A mindfulness-based social and emotional learning curriculum for school-aged children: The MindUP program. In Handbook of mindfulness in education: Integrating theory and research into practice. New York, NY: Springer New York. (pp. 313–334).

[ref67] Martin L. White M. P. Hunt A. Richardson M. Pahl S. Burt J. (2020). Nature contact, nature connectedness and associations with health, wellbeing and pro-environmental behaviours. J. Environ. Psychol. 68:101389. doi: 10.1016/j.jenvp.2020.101389

[ref68] Matiz A. Fabbro F. Crescentini C. (2024). Mindfulness through storytelling for mental health of primary school children: impact on acceptability and its associations with personality. Psychol. Res. Behav. Manag. 17, 1757–1774. doi: 10.2147/PRBM.S441494, PMID: 38686324 PMC11057635

[ref69] Mayer F. S. Frantz C. M. (2004). The connectedness to nature scale: a measure of individuals’ feeling in community with nature. J. Environ. Psychol. 24, 503–515. doi: 10.1016/j.jenvp.2004.10.001

[ref70] Mayer F. S. Frantz C. M. Bruehlman-Senecal E. Dolliver K. (2009). Why is nature beneficial? The role of connectedness to nature. Environ. Behav. 41, 607–643. doi: 10.1177/0013916508319745

[ref71] McKeering P. Hwang Y. S. (2019). A systematic review of mindfulness-based school interventions with early adolescents. Mindfulness 10, 593–610. doi: 10.1007/s12671-018-0998-9

[ref72] Narváez Carrión C. R. Luna Guillén A. P. Rosales Cevallos M. M. (2024). Perspective chapter: loneliness through time – geographical distance, sensibility isolation, and Hyperconnectivity. In Z. Ahmed, Determinants of Loneliness. IntechOpen. Available online at: https://www.intechopen.com/chapters/1173547 (Accessed April 10, 2025)., PMID: 32864128

[ref73] Nisbet E. Zelenski J. M. Grandpierre Z. (2019). Mindfulness in nature enhances connectedness and mood. Ecopsychology 11, 81–91. doi: 10.1089/eco.2018.0061

[ref74] O’Brien K. (2022). Systems change as “response-ability”. Soc. Innovations J. 15. Available online at: https://socialinnovationsjournal.com/index.php/sij/article/view/4997 (Accessed April 3, 2025).

[ref75] Oh R. R. Y. Fielding K. S. Nghiem L. T. P. Chang C. C. Carrasco L. R. Fuller R. A. (2021). Connection to nature is predicted by family values, social norms and personal experiences of nature. Global Ecol. Conservation 28:e01632. doi: 10.1016/j.gecco.2021.e01632

[ref76] Phan M. L. Renshaw T. L. Caramanico J. Greeson J. M. MacKenzie E. Atkinson-Diaz Z.. (2022). Mindfulness-based school interventions: a systematic review of outcome evidence quality by study design. Mindfulness 13, 1591–1613. doi: 10.1007/s12671-022-01885-9, PMID: 36186722 PMC9524483

[ref77] Primasari A. Yuniarti K. (2021). Enjoying every moment: improving adolescent’s subjective well-being through adolescent mindfulness program. Gadjah Mada J. Professional Psychol. (GamaJPP) 7, 115–128. doi: 10.22146/gamajpp.65594

[ref78] Quinto R. M. Russo F. Scafuto F. Innamorati M. Montecucco F. N. Ghiroldi S. (2025). Effects of a body-based mindfulness program on alexithymia, dispositional mindfulness, and distress symptoms: a pilot clinical trial. Behav. Sci. 15:55. doi: 10.3390/bs15010055, PMID: 39851859 PMC11763314

[ref79] R Core Team (2022). R: A language and environment for statistical computing. Vienna: R Foundation for Statistical Computing.

[ref80] Rasbash J. Steele F. Browne W. J. Goldstein H. (2009). User's guide to MlwiN Centre for Multilevel modelling. United Kingdom: University of Bristol.

[ref81] Reangsing C. Punsuwun S. Schneider J. K. (2020). Effects of mindfulness interventions on depressive symptoms in adolescents: a meta-analysis. Int. J. Nurs. Stud. 115. doi: 10.1016/j.ijnurstu.2020.103848 33383273

[ref82] Richardson M. Passmore H. A. Lumber R. Thomas R. Hunt A. (2021). Moments, not minutes: the nature-wellbeing relationship. Int. J. Wellbeing 11, 8–33. doi: 10.5502/ijw.v11i1.1267

[ref83] Roeser R. W. Pinela C. (2014). Mindfulness and compassion training in adolescence: a developmental contemplative science perspective. New Dir. Youth Dev. 2014, 9–30. doi: 10.1002/yd.20094, PMID: 25100492

[ref84] Ryan R. M. Deci E. L. (2001). On happiness and human potentials: a review of research on hedonic and eudaimonic wellbeing. Annu. Rev. Psychol. 52, 141–166. doi: 10.1146/annurev.psych.52.1.141, PMID: 11148302

[ref85] Ryff C. D. Keyes C. L. (1995). The structure of psychological wellbeing revisited. J. Pers. Soc. Psychol. 69, 719–727. 7473027 10.1037//0022-3514.69.4.719

[ref86] Scafuto F. (2019). Climate risk for the self and community: the role of nature relatedness, personal control and mindfulness. Riv. Stud. Sosten. 2, 89–108. doi: 10.3280/riss2019-002008

[ref87] Scafuto F. (2021). Individual and social-psychological factors to explain climate change efficacy: the role of mindfulness, sense of global community, and egalitarianism. J. Community Psychol. 49, 2003–2022. doi: 10.1002/jcop.22576, PMID: 33855729

[ref88] Scafuto F. Ciacchini R. Orrù G. Crescentini C. Conversano C. Mastorci F.. (2023). COVID-19 pandemic and internet addiction in young adults: a pilot study on positive and negative psychosocial correlates. Clin. Neuropsychiatry 20, 240–251. doi: 10.36131/cnfioritieditore20230403, PMID: 37791079 PMC10544255

[ref89] Scafuto F. Crescentini C. Conversano C. Matiz A. De Vincenzo F. Ghiroldi S.. (2025). Enhancing universal values through mindfulness and psychological well-being: a randomized-controlled trial with adolescents. Child Youth Care Forum. doi: 10.1007/s10566-025-09871-7

[ref90] Scafuto F. Ghiroldi S. Montecucco N. F. De Vincenzo F. Quinto R. M. Presaghi F.. (2024a). Promoting well-being in early adolescents through mindfulness: a cluster randomized controlled trial. J. Adolesc. 96, 57–69. doi: 10.1002/jad.12252, PMID: 37740437

[ref91] Scafuto F. Ghiroldi S. Montecucco N. F. Presaghi F. (2022). The mindfulness-based Gaia program reduces internalizing problems in high-school adolescents: a cluster randomized controlled trial. Mindfulness 13, 1804–1815. doi: 10.1007/s12671-022-01920-9

[ref92] Scafuto F. La Barbera F. (2016). Protest against waste contamination in the ‘land of fires’: psychological antecedents for activists and non-activists. J. Community Appl. Soc. Psychol. 26, 481–495. doi: 10.1177/10888683231178056

[ref93] Scafuto F. Quinto R. M. Ghiroldi S. Montecucco N. F. Presaghi F. Iani L.. (2024b). The mediation role of emotion regulation strategies on the relationship between mindfulness effects, psychological well-being and distress among youths: findings from a randomized controlled trial. Curr. Psychol. 43, 24295–24307. doi: 10.1007/s12144-024-06081-7

[ref94] Schreier M. (2012). Qualitative content analysis in practice. UK: SAGE Publications Ltd.

[ref95] Schultz P. W. (2002). “Inclusion with nature: the psychology of human-nature relations” in Psychology of sustainable development. eds. Schmuck P. Schultz P. W. (New York: Springer), 61–78.

[ref96] Schutte N. S. Malouff J. M. (2018). Mindfulness and connectedness to nature: a meta-analytic investigation. Pers. Individ. Differ. 127, 10–14. doi: 10.1016/j.paid.2018.01.034

[ref97] Sheffield D. Butler C. W. Richardson M. (2022). Improving nature connectedness in adults: a meta-analysis, review and agenda. Sustainability 14:12494. doi: 10.3390/su141912494

[ref98] Sigmon S. T. Whitcomb S. R. Snyder C. R. (2002). “Psychological Home” in Psychological sense of community. eds. Fisher A. T. Sonn C. C. Bishop B. J. (Boston, MA: Springer), 25–41.

[ref99] Sirigatti S. Penzo I. Iani L. Mazzeschi A. Hatalskaja H. Giannetti E.. (2013). Measurement invariance of Ryff’s psychological wellbeing scales across Italian and Belarusian students. Soc. Indic. Res. 113, 67–80. doi: 10.1007/s11205-012-0082-0

[ref100] Tang Y.-Y. Tang R. Gross J. J. (2019). Promoting psychological well-being through an evidence-based mindfulness training program. Front. Hum. Neurosci. 13:237. doi: 10.3389/fnhum.2019.00237, PMID: 31354454 PMC6635568

[ref101] Thiermann U. B. Sheate W. R. (2020). The way forward in mindfulness and sustainability: a critical review and research agenda. J. Cogn. Enhanc. 5, 118–139. doi: 10.1007/s41465-020-00180-6

[ref102] Thomson E. E. Roach S. P. (2023). The relationships among nature connectedness, climate anxiety, climate action, climate knowledge, and mental health. Front. Psychol. 14:1241400. doi: 10.3389/fpsyg.2023.1241400, PMID: 38034293 PMC10684686

[ref103] Ulrich R. S. Simons R. F. Losito B. D. Fiorito E. Miles M. A. Zelson M. (1991). Stress recovery during exposure to natural and urban environments. J. Environ. Psychol. 11, 201–230.

[ref105] Unsworth S. Palicki S. K. Lustig J. (2016). The impact of mindful meditation in nature on self-nature interconnectedness. Mindfulness 7, 1052–1060. doi: 10.1007/s12671-016-0542-8

[ref106] Veltri A. Scarpellini P. Piccinni A. Conversano C. Giacomelli C. Bombardieri S.. (2012). Methodological approach to depressive symptoms in fibromyalgia patients. Clin. Exp. Rheumatol. 30, 136–142. 23261013

[ref107] Viejo C. Gómez-López M. Ortega-Ruiz R. (2018). Adolescents’ psychological well-being: a multidimensional measure. Int. J. Environ. Res. Public Health 15:2325. doi: 10.3390/ijerph15102325, PMID: 30360456 PMC6210683

[ref108] Volanen S. M. Lassander M. Hankonen N. Santalahti P. Hintsanen M. Simonsen. (2020). Healthy learning mind–effectiveness of a mindfulness program on mental health compared to a relaxation program and teaching as usual in schools: a cluster-randomised controlled trial. J. Affect. Disord. 260, 660–669. doi: 10.1016/j.jad.2019.08.087 31546105

[ref109] Wamsler C. Brink E. (2018). Mindsets for sustainability: exploring the link between mindfulness and sustainable climate adaptation. Ecol. Econ. 151, 55–61. doi: 10.1016/j.ecolecon.2018.04.029

[ref110] Wang J. Geng L. Schultz P. W. Zhou K. (2019). Mindfulness increases the belief in climate change: the mediating role of connectedness with nature. Environ. Behav. 51, 3–23. doi: 10.1177/0013916517738036

[ref111] Weil L. G. Fleming S. M. Dumontheil I. Kilford E. J. Blakemore S. J. (2013). The development of metacognitive ability in adolescence. Conscious. Cogn. 22, 264–271. doi: 10.1016/j.concog.2013.01.004, PMID: 23376348 PMC3719211

[ref104] WHO & UNICEF (2023). Global report on children with developmental disabilities: From the margins to the mainstream. Geneva: World Health Organization. Available online at https://www.unicef.org/media/145016/file/Global-report-on-children-withdevelopmental-disabilities-2023.pdf (Accessed April 3, 2025).

[ref112] Wilson E. O. (2002). The future of life. New York: Knopf.

[ref113] Yeager D. S. Bryan C. J. Gross J. J. Murray J. S. Krettek Cobb D. Santos P. H. F.. (2022). A synergistic mindsets intervention protects adolescents from stress. Nature 607, 512–520. doi: 10.1038/s41586-022-04907-7, PMID: 35794485 PMC9258473

[ref114] Yousefi Afrashteh M. Hasani F. (2022). Mindfulness and psychological wellbeing in adolescents: the mediating role of self-compassion, emotional dysregulation, and cognitive flexibility. Borderline Personality Disord. Emotion Dysregulation 9:22. doi: 10.1186/s40479-022-00192-y, PMID: 36059027 PMC9441221

[ref115] Zhang D. Lee E. K. Mak E. C. Ho C. Y. Wong S. Y. (2021). Mindfulness-based interventions: an overall review. Br. Med. Bull. 138, 41–57. doi: 10.1093/bmb/ldab005, PMID: 33884400 PMC8083197

[ref116] Zhang Z. Parker R. M. A. Charlton C. M. J. Leckie G. Browne W. J. (2016). R2MLwiN: a package to run MLwiN from within R. J. Stat. Softw. 72, 1–43. doi: 10.18637/jss.v072.i10

