# Supplementary material for: MINDhEARTH: a school-based intervention to improve personal well-being, mindfulness and connectedness to nature in adolescents
Source: Front Psychol. 2025 Sep 8;16:1628048. doi: 10.3389/fpsyg.2025.1628048 (PMC12450908; doi:10.3389/fpsyg.2025.1628048)
Supplement: Supplementary file 1 [file Table_1.docx]

Table S1 – Descriptive statistics (M and SD) at pre- and post-test as a function of the intervention for all outcomes.

|  | *Pre-test* | | | | | | | | | | | | | | | | Post-test | | | | | | | | | | | | | | | |
| --- | --- | --- | --- | --- | --- | --- | --- | --- | --- | --- | --- | --- | --- | --- | --- | --- | --- | --- | --- | --- | --- | --- | --- | --- | --- | --- | --- | --- | --- | --- | --- | --- |
|  | 1st year high school | | | | *2nd year high school* | | | | *3rd year high school* | | | | 4th year high school | | | | 1st year high school | | | | *2nd year high school* | | | | *3rd year high school* | | | | 4th year high school | | | |
|  | *Control* | | *Intervention* | | *Control* | | *Intervention* | | *Control* | | *Intervention* | | *Control* | | *Intervention* | | *Control* | | *Intervention* | | *Control* | | *Intervention* | | *Control* | | *Intervention* | | *Control* | | *Intervention* | |
|  | *M* | *SD* | *M* | *SD* | *M* | *SD* | *M* | *SD* | *M* | *SD* | *M* | *SD* | *M* | *SD* | *M* | *SD* | *M* | *SD* | *M* | *SD* | *M* | *SD* | *M* | *SD* | *M* | *SD* | *M* | *SD* | *M* | *SD* | *M* | *SD* |
| SH | 3.85 | 1.04 | 3.60 | 1.04 | 3.82 | 0.97 | 4.04 | 0.80 | 3.84 | 1.20 | 3.71 | 1.36 | 3.75 | 0.60 | 3.93 | 1.05 | 3.78 | 1.03 | 3.86 | 1.15 | 3.78 | 0.88 | 4.34 | 0.81 | 3.71 | 1.12 | 3.92 | 0.77 | 3.78 | 0.59 | 4.03 | 1.11 |
| NC | 3.06 | 0.46 | 2.74 | 0.57 | 2.99 | 0.55 | 2.82 | 0.54 | 3.29 | 0.48 | 3.02 | 0.67 | 3.21 | 0.68 | 3.13 | 0.67 | 3.06 | 0.52 | 2.82 | 0.61 | 3.16 | 0.59 | 3.12 | 0.75 | 3.15 | 0.49 | 3.15 | 0.22 | 3.32 | 0.45 | 3.24 | 0.60 |
| FFMQ-O | 2.64 | 0.89 | 2.08 | 0.92 | 2.57 | 0.94 | 2.42 | 0.95 | 2.49 | 0.94 | 2.75 | 0.93 | 2.81 | 0.88 | 2.83 | 0.71 | 2.42 | 0.90 | 2.30 | 1.04 | 2.78 | 0.87 | 2.55 | 1.02 | 3.02 | 0.90 | 2.50 | 0.72 | 3.11 | 0.58 | 2.51 | 0.91 |
| FFMQ-D | 2.60 | 0.72 | 2.57 | 0.77 | 2.65 | 0.62 | 2.93 | 0.93 | 2.69 | 0.89 | 2.50 | 0.81 | 2.93 | 0.74 | 2.89 | 0.64 | 2.54 | 0.89 | 2.80 | 0.90 | 2.77 | 0.63 | 3.10 | 0.63 | 2.75 | 0.71 | 2.28 | 0.65 | 2.83 | 0.87 | 3.08 | 0.59 |
| FFMQ-A | 3.75 | 0.72 | 3.77 | 1.04 | 3.48 | 0.97 | 3.53 | 0.85 | 3.59 | 0.89 | 3.94 | 1.14 | 3.44 | 1.08 | 3.94 | 0.51 | 3.25 | 0.86 | 4.14 | 0.62 | 3.61 | 0.67 | 3.64 | 0.92 | 3.35 | 0.78 | 4.03 | 0.70 | 2.81 | 0.87 | 3.84 | 0.66 |
| FFMQ-NJ | 3.22 | 1.12 | 3.55 | 1.10 | 3.42 | 0.97 | 3.63 | 0.88 | 3.00 | 0.99 | 2.78 | 0.89 | 3.56 | 0.73 | 2.97 | 0.90 | 3.54 | 0.82 | 3.72 | 1.07 | 3.41 | 0.74 | 3.75 | 0.85 | 3.02 | 0.88 | 3.50 | 1.17 | 3.39 | 0.79 | 3.91 | 1.00 |
| FFMQ-NR | 2.72 | 0.76 | 2.51 | 0.97 | 2.38 | 0.81 | 2.41 | 0.82 | 2.55 | 0.87 | 2.28 | 0.44 | 2.37 | 0.98 | 2.89 | 0.58 | 2.56 | 0.74 | 2.05 | 0.80 | 2.49 | 0.79 | 2.38 | 0.88 | 2.68 | 0.70 | 2.44 | 0.62 | 2.57 | 0.79 | 2.42 | 0.75 |
| PWB-SA | 3.64 | 1.21 | 3.29 | 1.03 | 3.75 | 0.94 | 3.61 | 0.94 | 3.84 | 0.96 | 4.11 | 1.24 | 3.56 | 0.44 | 3.47 | 1.22 | 3.68 | 1.06 | 3.59 | 1.29 | 3.68 | 0.86 | 3.63 | 1.04 | 3.76 | 1.07 | 3.33 | 1.40 | 3.52 | 0.67 | 3.76 | 1.10 |
| PWB-A | 4.04 | 0.78 | 3.80 | 0.80 | 4.00 | 0.72 | 3.92 | 0.99 | 4.35 | 0.98 | 4.33 | 1.12 | 4.04 | 0.84 | 3.98 | 1.13 | 3.94 | 0.84 | 4.07 | 0.75 | 3.88 | 0.82 | 4.06 | 0.86 | 4.25 | 0.76 | 4.06 | 0.39 | 3.85 | 0.93 | 4.27 | 0.80 |
| PWB-EM | 3.67 | 1.05 | 3.71 | 0.91 | 3.58 | 0.94 | 3.72 | 1.02 | 3.78 | 0.89 | 3.94 | 0.98 | 3.41 | 0.94 | 3.84 | 0.73 | 3.80 | 0.95 | 3.81 | 0.86 | 3.72 | 0.84 | 3.87 | 0.87 | 3.96 | 0.82 | 4.22 | 0.54 | 3.59 | 1.28 | 3.67 | 0.76 |
| PWB-PG | 4.25 | 0.81 | 3.58 | 1.06 | 4.31 | 0.79 | 4.23 | 0.96 | 4.67 | 0.84 | 4.78 | 0.86 | 4.44 | 0.67 | 4.62 | 0.87 | 4.21 | 0.93 | 3.84 | 1.27 | 4.62 | 0.79 | 4.00 | 1.27 | 4.51 | 1.12 | 3.72 | 0.83 | 4.07 | 0.81 | 4.33 | 0.90 |
| PWB-PR | 4.35 | 1.01 | 3.67 | 1.15 | 4.01 | 1.08 | 4.08 | 0.99 | 4.06 | 1.25 | 4.17 | 1.21 | 4.59 | 0.70 | 4.44 | 1.04 | 4.10 | 0.98 | 4.06 | 1.12 | 3.99 | 0.90 | 4.15 | 0.96 | 4.24 | 1.13 | 4.00 | 0.94 | 4.11 | 0.78 | 4.07 | 0.82 |
| PWB-PL | 3.56 | 0.89 | 3.59 | 0.95 | 3.37 | 0.99 | 3.40 | 1.01 | 3.92 | 0.76 | 4.22 | 0.66 | 3.52 | 0.99 | 3.62 | 0.79 | 3.27 | 0.94 | 3.46 | 0.80 | 3.43 | 0.96 | 3.80 | 1.00 | 3.49 | 1.07 | 3.83 | 0.91 | 3.44 | 1.35 | 3.62 | 0.82 |

Note: SH = Subjective Happiness; NC = Nature Connectedness; FFMQ-O = FFMQ Observing; FFMQ-D = FFMQ Describing; FFMQ-A = FFMQ Acting; FFMQ-NJ = FFMQ Non-judging; FFMQ-NR = FFMQ Non-reacting; PWB-SA = PWB Self-Acceptance; PWB-A = PWB Autonomy; PWB-EM = PWB Environmental Mastery; PWB-PG = PWB Personal Growth; PWB-PR = PWB Positive Relations; PWB-PL = PWB Purpose in Life
