# Supplementary material for: MINDhEARTH: a school-based intervention to improve personal well-being, mindfulness and connectedness to nature in adolescents
Source: Front Psychol. 2025 Sep 8;16:1628048. doi: 10.3389/fpsyg.2025.1628048 (PMC12450908; doi:10.3389/fpsyg.2025.1628048)
Supplement: Supplementary file 2 [file Table_2.docx]

Table S2 - Intervention efficacy for PWB Personal Growth

|  |  | *b* | *s.e.* | *p-value* | *L.L. 95% Cred. Int.* | *U.L. 95% Cred. Int.* |
| --- | --- | --- | --- | --- | --- | --- |
| Fixed effects: |  |  |  |  |  |  |
|  | Constant | 3.960 | 0.293 | 0.000 | 3.396 | 4.542 |
|  | Intervention | -0.225 | 0.148 | 0.127 | -0.516 | 0.062 |
|  | Time | 0.009 | 0.057 | 0.875 | -0.104 | 0.124 |
|  | Gender (Female) | 0.240 | 0.153 | 0.118 | -0.063 | 0.537 |
|  | Age | 0.203 | 0.077 | 0.009 | 0.054 | 0.358 |
|  | Intervention*Time | -0.087 | 0.083 | 0.293 | -0.251 | 0.073 |
| Random Effects: |  |  |  |  |  |  |
|  | L3-Classes: Constant | 0.016 | 0.043 |  | 0.001 | 0.094 |
|  | L2-Students: Constant | 0.433 | 0.088 |  | 0.277 | 0.620 |
|  | L1-Time: Constant | 2.461 | 2.796 |  | -2.633 | 7.078 |
|  | L1-Time: Constant*Time | 0.081 | 0.036 |  | 0.013 | 0.153 |
|  | L1-Time: Time | -1.938 | 2.805 |  | -6.569 | 3.181 |
| *Note: Model Fit D-bar = 649.21; L.L. 95% Cred. Int. = Lower Level Bayesian 95% Credible Interval; U.L. 95% Cred. Int. = Upper Level Bayesian 95% Credible Interval;* | | | | | | |
