# Supplementary material for: MINDhEARTH: a school-based intervention to improve personal well-being, mindfulness and connectedness to nature in adolescents
Source: Front Psychol. 2025 Sep 8;16:1628048. doi: 10.3389/fpsyg.2025.1628048 (PMC12450908; doi:10.3389/fpsyg.2025.1628048)
Supplement: Supplementary file 3 [file Table_3.docx]

Table S3 - Intervention efficacy for PWB Purpose in Life

|  |  | *b* | *s.e.* | *p-value* | *L.L. 95% Cred. Int.* | *U.L. 95% Cred. Int.* |
| --- | --- | --- | --- | --- | --- | --- |
| Fixed effects: |  |  |  |  |  |  |
|  | Constant | 3.762 | 0.307 | 0.000 | 3.157 | 4.358 |
|  | Intervention | 0.084 | 0.151 | 0.577 | -0.209 | 0.380 |
|  | Time | -0.088 | 0.048 | 0.067 | -0.181 | 0.007 |
|  | Gender (Female) | -0.161 | 0.160 | 0.315 | -0.475 | 0.155 |
|  | Age | 0.087 | 0.080 | 0.279 | -0.076 | 0.241 |
|  | Intervention*Time | 0.130 | 0.070 | 0.062 | -0.005 | 0.266 |
| Random Effects: |  |  |  |  |  |  |
|  | L3-Classes: Constant | 0.021 | 0.052 |  | 0.001 | 0.121 |
|  | L2-Students: Constant | 0.532 | 0.088 |  | 0.378 | 0.725 |
|  | L1-Time: Constant | 3.198 | 1.868 |  | 0.608 | 7.047 |
|  | L1-Time: Constant*Time | 0.019 | 0.031 |  | -0.041 | 0.082 |
|  | L1-Time: Time | -2.834 | 1.875 |  | -6.699 | -0.232 |
| *Note: Model Fit D-bar = 554.34; L.L. 95% Cred. Int. = Lower Level Bayesian 95% Credible Interval; U.L. 95% Cred. Int. = Upper Level Bayesian 95% Credible Interval;* | | | | | | |
