# Supplementary material for: MINDhEARTH: a school-based intervention to improve personal well-being, mindfulness and connectedness to nature in adolescents
Source: Front Psychol. 2025 Sep 8;16:1628048. doi: 10.3389/fpsyg.2025.1628048 (PMC12450908; doi:10.3389/fpsyg.2025.1628048)
Supplement: Supplementary file 4 [file Table_4.docx]

Table S4 - Intervention efficacy for PWB Environmental Mastery

|  |  | *b* | *s.e.* | *p-value* | *L.L. 95% Cred. Int.* | *U.L. 95% Cred. Int.* |
| --- | --- | --- | --- | --- | --- | --- |
| Fixed effects: |  |  |  |  |  |  |
|  | Constant | 3.984 | 0.293 | <.001 | 3.402 | 4.561 |
|  | Intervention | 0.044 | 0.147 | 0.767 | -0.237 | 0.338 |
|  | Time | 0.076 | 0.045 | 0.093 | -0.012 | 0.163 |
|  | Gender (Female) | -0.159 | 0.154 | 0.302 | -0.460 | 0.142 |
|  | Age | 0.053 | 0.078 | 0.500 | -0.099 | 0.211 |
|  | Intervention*Time | -0.036 | 0.065 | 0.576 | -0.165 | 0.092 |
| Random Effects: |  |  |  |  |  |  |
|  | L3-Classes: Constant | 0.018 | 0.049 |  | 0.001 | 0.103 |
|  | L2-Students: Constant | 0.524 | 0.084 |  | 0.376 | 0.706 |
|  | L1-Time: Constant | -0.373 | 1.496 |  | -3.625 | 2.190 |
|  | L1-Time: Constant*Time | -0.030 | 0.028 |  | -0.089 | 0.025 |
|  | L1-Time: Time | 0.698 | 1.501 |  | -1.867 | 3.945 |
| *Note: Model Fit D-bar = 505.47; L.L. 95% Cred. Int. = Lower Level Bayesian 95% Credible Interval; U.L. 95% Cred. Int. = Upper Level Bayesian 95% Credible Interval;* | | | | | | |
