# Supplementary material for: MINDhEARTH: a school-based intervention to improve personal well-being, mindfulness and connectedness to nature in adolescents
Source: Front Psychol. 2025 Sep 8;16:1628048. doi: 10.3389/fpsyg.2025.1628048 (PMC12450908; doi:10.3389/fpsyg.2025.1628048)
Supplement: Supplementary file 5 [file Table_5.docx]

Table S5 - Intervention efficacy for PWB Positive Relations

|  |  | *b* | *s.e.* | *p-value* | *L.L. 95% Cred. Int.* | *U.L. 95% Cred. Int.* |
| --- | --- | --- | --- | --- | --- | --- |
| Fixed effects: |  |  |  |  |  |  |
|  | Constant | 4.077 | 0.318 | 0.000 | 3.451 | 4.699 |
|  | Intervention | -0.073 | 0.160 | 0.648 | -0.384 | 0.239 |
|  | Time | -0.055 | 0.055 | 0.316 | -0.162 | 0.050 |
|  | Gender (Female) | 0.040 | 0.168 | 0.811 | -0.289 | 0.367 |
|  | Age | 0.042 | 0.083 | 0.614 | -0.125 | 0.203 |
|  | Intervention*Time | 0.084 | 0.079 | 0.284 | -0.071 | 0.240 |
| Random Effects: |  |  |  |  |  |  |
|  | L3-Classes: Constant | 0.014 | 0.030 |  | 0.001 | 0.077 |
|  | L2-Students: Constant | 0.599 | 0.104 |  | 0.415 | 0.820 |
|  | L1-Time: Constant | 1.641 | 1.759 |  | -1.533 | 5.081 |
|  | L1-Time: Constant*Time | -0.056 | 0.038 |  | -0.133 | 0.018 |
|  | L1-Time: Time | -1.168 | 1.764 |  | -4.639 | 2.008 |
| *Note: Model Fit D-bar = 622.25; L.L. 95% Cred. Int. = Lower Level Bayesian 95% Credible Interval; U.L. 95% Cred. Int. = Upper Level Bayesian 95% Credible Interval;* | | | | | | |
