# Supplementary material for: MINDhEARTH: a school-based intervention to improve personal well-being, mindfulness and connectedness to nature in adolescents
Source: Front Psychol. 2025 Sep 8;16:1628048. doi: 10.3389/fpsyg.2025.1628048 (PMC12450908; doi:10.3389/fpsyg.2025.1628048)
Supplement: Supplementary file 6 [file Table_6.docx]

Table S6 - Intervention efficacy for PWB Self-Acceptance

|  |  | *b* | *s.e.* | *p* | *L.L. 95% Cred. Int.* | *U.L. 95% Cred. Int.* |
| --- | --- | --- | --- | --- | --- | --- |
| Fixed effects: |  |  |  |  |  |  |
|  | Constant | 3.959 | 0.346 | <.001 | 3.264 | 4.624 |
|  | Intervention | -0.184 | 0.168 | 0.274 | -0.519 | 0.141 |
|  | Time | -0.017 | 0.051 | 0.744 | -0.116 | 0.085 |
|  | Gender (Female) | -0.148 | 0.181 | 0.414 | -0.498 | 0.215 |
|  | Age | 0.180 | 0.102 | 0.077 | 0.000 | 0.407 |
|  | Intervention*Time | 0.067 | 0.075 | 0.369 | -0.081 | 0.211 |
| Random Effects: |  |  |  |  |  |  |
|  | L3-Classes: Constant | 0.034 | 0.095 |  | 0.001 | 0.233 |
|  | L2-Students: Constant | 0.663 | 0.106 |  | 0.476 | 0.891 |
|  | L1-Time: Constant | 1.857 | 2.025 |  | -1.773 | 6.252 |
|  | L1-Time: Constant*Time | 0.020 | 0.036 |  | -0.052 | 0.092 |
|  | L1-Time: Time | -1.440 | 2.032 |  | -5.838 | 2.208 |
| *Note: Model Fit D-bar = 591.87L.L. 95% Cred. Int. = Lower Level Bayesian 95% Credible Interval; U.L. 95% Cred. Int. = Upper Level Bayesian 95% Credible Interval;* | | | | | | |
