# Supplementary material for: MINDhEARTH: a school-based intervention to improve personal well-being, mindfulness and connectedness to nature in adolescents
Source: Front Psychol. 2025 Sep 8;16:1628048. doi: 10.3389/fpsyg.2025.1628048 (PMC12450908; doi:10.3389/fpsyg.2025.1628048)
Supplement: Supplementary file 7 [file Table_7.docx]

Table S7 - Intervention efficacy for FFMQ Non-Reacting

|  |  | *b* | *s.e.* | *p-value* | *L.L. 95% Cred. Int.* | *U.L. 95% Cred. Int.* |
| --- | --- | --- | --- | --- | --- | --- |
| Fixed effects: |  |  |  |  |  |  |
|  | Constant | 2.353 | 0.237 | 0.000 | 1.892 | 2.829 |
|  | Intervention | -0.081 | 0.115 | 0.483 | -0.308 | 0.146 |
|  | Time | 0.016 | 0.054 | 0.758 | -0.091 | 0.121 |
|  | Gender (Female) | 0.106 | 0.123 | 0.387 | -0.137 | 0.345 |
|  | Age | 0.081 | 0.067 | 0.227 | -0.042 | 0.218 |
|  | Intervention*Time | -0.137 | 0.078 | 0.078 | -0.289 | 0.017 |
| Random Effects: |  |  |  |  |  |  |
|  | L3-Classes: Constant | 0.018 | 0.040 |  | 0.001 | 0.107 |
|  | L2-Students: Constant | 0.195 | 0.059 |  | 0.085 | 0.315 |
|  | L1-Time: Constant | -0.259 | 2.273 |  | -4.749 | 4.344 |
|  | L1-Time: Constant*Time | -0.020 | 0.026 |  | -0.073 | 0.031 |
|  | L1-Time: Time | 0.716 | 2.281 |  | -3.912 | 5.226 |
| *Note: Model Fit D-bar = 617.77; L.L. 95% Cred. Int. = Lower Level Bayesian 95% Credible Interval; U.L. 95% Cred. Int. = Upper Level Bayesian 95% Credible Interval;* | | | | | | |
