# Supplementary material for: MINDhEARTH: a school-based intervention to improve personal well-being, mindfulness and connectedness to nature in adolescents
Source: Front Psychol. 2025 Sep 8;16:1628048. doi: 10.3389/fpsyg.2025.1628048 (PMC12450908; doi:10.3389/fpsyg.2025.1628048)
Supplement: Supplementary file 8 [file Table_8.docx]

Table S8- Intervention efficacy for FFMQ Observing

|  |  | *b* | *s.e.* | *p-value* | *L.L. 95% Cred. Int.* | *U.L. 95% Cred. Int.* |
| --- | --- | --- | --- | --- | --- | --- |
| Fixed effects: |  |  |  |  |  |  |
|  | Constant | 2.239 | 0.285 | 0.000 | 1.690 | 2.811 |
|  | Intervention | -0.122 | 0.133 | 0.357 | -0.384 | 0.138 |
|  | Time | 0.072 | 0.058 | 0.214 | -0.041 | 0.186 |
|  | Gender (Female) | 0.242 | 0.146 | 0.098 | -0.051 | 0.524 |
|  | Age | 0.159 | 0.082 | 0.052 | 0.005 | 0.332 |
|  | Intervention*Time | -0.055 | 0.084 | 0.515 | -0.219 | 0.109 |
| Random Effects: |  |  |  |  |  |  |
|  | L3-Classes: Constant | 0.041 | 0.080 |  | 0.001 | 0.214 |
|  | L2-Students: Constant | 0.284 | 0.078 |  | 0.141 | 0.445 |
|  | L1-Time: Constant | -2.217 | 2.512 |  | -7.574 | 2.016 |
|  | L1-Time: Constant*Time | 0.017 | 0.033 |  | -0.047 | 0.081 |
|  | L1-Time: Time | 2.761 | 2.518 |  | -1.483 | 8.140 |
| *Note: Model Fit D-bar = 667.49; L.L. 95% Cred. Int. = Lower Level Bayesian 95% Credible Interval; U.L. 95% Cred. Int. = Upper Level Bayesian 95% Credible Interval;* | | | | | | |
