# Supplementary material for: MINDhEARTH: a school-based intervention to improve personal well-being, mindfulness and connectedness to nature in adolescents
Source: Front Psychol. 2025 Sep 8;16:1628048. doi: 10.3389/fpsyg.2025.1628048 (PMC12450908; doi:10.3389/fpsyg.2025.1628048)
Supplement: Supplementary file 9 [file Table_9.docx]

Table S9 - Intervention efficacy for FFMQ Describing

|  |  | *b* | *s.e.* | *p-value* | *L.L. 95% Cred. Int.* | *U.L. 95% Cred. Int.* |
| --- | --- | --- | --- | --- | --- | --- |
| Fixed effects: |  |  |  |  |  |  |
|  | Constant | 2.759 | 0.240 | 0.000 | 2.286 | 3.223 |
|  | Intervention | 0.135 | 0.118 | 0.250 | -0.098 | 0.365 |
|  | Time | 0.011 | 0.043 | 0.791 | -0.073 | 0.097 |
|  | Gender (Female) | -0.038 | 0.126 | 0.760 | -0.286 | 0.208 |
|  | Age | 0.128 | 0.066 | 0.053 | -0.000 | 0.263 |
|  | Intervention*Time | 0.068 | 0.063 | 0.276 | -0.055 | 0.191 |
| Random Effects: |  |  |  |  |  |  |
|  | L3-Classes: Constant | 0.016 | 0.034 |  | 0.001 | 0.090 |
|  | L2-Students: Constant | 0.286 | 0.056 |  | 0.187 | 0.407 |
|  | L1-Time: Constant | -0.776 | 1.236 |  | -2.878 | 1.607 |
|  | L1-Time: Constant*Time | -0.005 | 0.022 |  | -0.047 | 0.038 |
|  | L1-Time: Time | 1.084 | 1.240 |  | -1.305 | 3.197 |
| *Note: Model Fit D-bar = 494.95; L.L. 95% Cred. Int. = Lower Level Bayesian 95% Credible Interval; U.L. 95% Cred. Int. = Upper Level Bayesian 95% Credible Interval;* | | | | | | |
