# Supplementary material for: MINDhEARTH: a school-based intervention to improve personal well-being, mindfulness and connectedness to nature in adolescents
Source: Front Psychol. 2025 Sep 8;16:1628048. doi: 10.3389/fpsyg.2025.1628048 (PMC12450908; doi:10.3389/fpsyg.2025.1628048)
Supplement: Supplementary file 10 [file Table_10.docx]

Table S10 - Intervention efficacy for FFMQ Non-judging

|  |  | *b* | *s.e.* | *p-value* | *L.L. 95% Cred. Int.* | *U.L. 95% Cred. Int.* |
| --- | --- | --- | --- | --- | --- | --- |
| Fixed effects: |  |  |  |  |  |  |
|  | Constant | 3.393 | 0.310 | 0.000 | 2.756 | 3.990 |
|  | Intervention | 0.190 | 0.142 | 0.183 | -0.089 | 0.468 |
|  | Time | 0.045 | 0.057 | 0.430 | -0.067 | 0.157 |
|  | Gender (Female) | -0.030 | 0.162 | 0.851 | -0.343 | 0.298 |
|  | Age | -0.015 | 0.088 | 0.865 | -0.181 | 0.171 |
|  | Intervention*Time | 0.134 | 0.082 | 0.104 | -0.027 | 0.294 |
| Random Effects: |  |  |  |  |  |  |
|  | L3-Classes: Constant | 0.057 | 0.113 |  | 0.001 | 0.306 |
|  | L2-Students: Constant | 0.375 | 0.083 |  | 0.225 | 0.551 |
|  | L1-Time: Constant | 2.863 | 4.264 |  | -2.634 | 16.138 |
|  | L1-Time: Constant*Time | -0.045 | 0.035 |  | -0.117 | 0.023 |
|  | L1-Time: Time | -2.345 | 4.278 |  | -15.669 | 3.165 |
| *Note: Model Fit D-bar = 657.91; L.L. 95% Cred. Int. = Lower Level Bayesian 95% Credible Interval; U.L. 95% Cred. Int. = Upper Level Bayesian 95% Credible Interval;* | | | | | | |
